# Deciphering coulombic loss in lithium-ion batteries and beyond

**DOI:** 10.1038/s41467-025-60833-y

**Published:** 2025-07-01

**Authors:** Jiyu Cai, Steve E. Trask, Zhenzhen Yang, Yingying Xie, Wenquan Lu, Hoai Nguyen, Yuzi Liu, Xiangbo Meng, Gabriel M. Veith, Hao Jia, Wu Xu, Zonghai Chen

**Affiliations:** 1 Chemcial Sciences and Engineering Division, Argonne National Laboratory, Lemont, IL USA; 2 Center for Nanoscale Materials, Argonne National Laboratory, Lemont, IL USA; 3 Department of Mechanical Engineering, University of Arkansas, Arkansas, AR USA; 4 Chemical Sciences Division, Oak Ridge National Laboratory, Oak Ridge, TN USA; 5 Energy and Environmental Directorate, Pacific Northwest National Laboratory, Richland, WA USA

**Keywords:** Batteries, Batteries, Energy

## Abstract

Lithium-ion batteries are pivotal for modern energy storage, yet accurately predicting their lifespan remains a critical challenge. While descriptors like coulombic efficiency are widely used to assess battery longevity, the unclear physical origins of coulombic losses cause semi-quantitative correlation with capacity, complicating battery development. Here, we combine high-precision leakage current and open-circuit-voltage measurements with charge conservation principles to explore microscopic charge consumptions at electrode-electrolyte interfaces across diverse chemistries. We demonstrate that coulombic loss arises from a synergy between local charge neutrality and global charge compensation, reconciling its quantitative correlation to capacity. Contrary to conventional assumptions equating coulombic loss with irreversible capacity loss, this framework resolves systematic overestimations and paradoxical phenomena in existing chemistries. Our findings establish physics-informed criteria for accelerated lifespan evaluation and guide rational design of long-life lithium-ion batteries and beyond.

## Abstract (web summary)

Conventional electrochemical evaluations severely decelerate the iteration of long-life batteries. Here, the authors unpuzzle the myth of coulombic loss contrary to prevailing understanding. Physical-correlated coulombic-capacity relation is pivotal for rapid battery screening and prognostics.

## Introduction

The global deployment of lithium-ion batteries (LIBs) in automobile applications critically demands higher energy density and longer lifespan^1–3^. Nevertheless, the current understanding of battery deterioration mechanisms and estimated lifetime leaves a technological bottleneck. The evaluation of novel LIB chemistries still depends on traditionally lengthy electrochemical characterizations like the charging/discharging cycling, but it becomes a practically infeasible task for real operation conditions like 1000 cycles and 7 calendar years for automobile batteries^4–6^. Advanced numerical approaches, like data-driven machine learning, have shown great capability for data-mining the underlying knowledge for performance evaluation, but still substantially depend on massive semi-empirical data for establishing necessary modality and dimension to learn the physical insights^7–10^. The cutting-edge characterization techniques are unrivaled in providing physical observations to understand degradation mechanisms in LIBs, but are limited to lengthy preparation and less accessibility for evaluating novel chemistries in the accelerated developments among academics and industries. In this context, it is essentially important to invest the community-wide efforts in advancing the physical understanding of LIB chemistries for establishing accurate performance evaluation and prognostics.

**Fig. 1:**
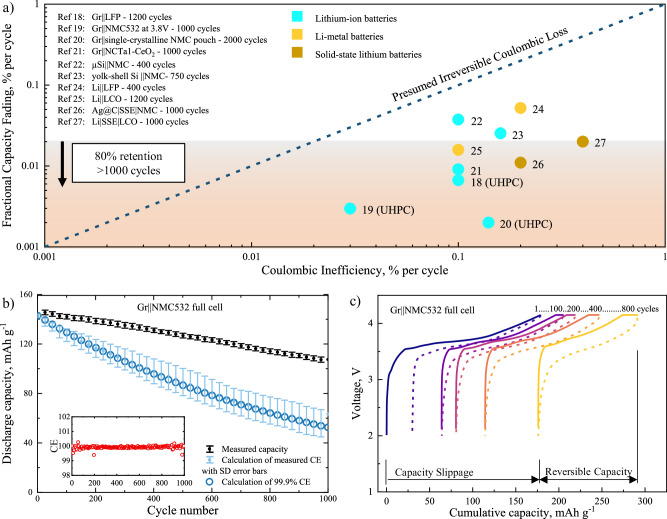
Notable inconsistency between the measured coulombic loss and capacity fading. **a** The overestimations of coulombic loss in CIE towards factional capacity fading per cycle among recently reported long-life liquid-electrolyte lithium-ion/lithium-metal batteries and solid-state-electrolyte (SSE) lithium batteries, no matter whether using high-precision measurements like UHPC or the optimized chemistries^18–27^. The black dashed line represents the prevailing belief of equivalent relationship based on the irreversible coulombic loss. **b** Cycling discharge capacity of Gr||NMC532 cell in the voltage range of 2.5–4.2 V at a constant current of C/2 (1.36 mA), showing the overestimation of irreversible charge inventory loss using measured and reported CE values. The measured capacity and CE-calculated capacity show the mean value of three repeating cells in the center with standard deviation (SD) error bars. **c** The voltage-capacity slippage of Gr||NMC532 cell during cycling, presenting non-equivalence of reversible capacity and the accumulated coulombic consumption by parasitic reactions.

Quantifying the amount of usable chemical energy in LIBs is practically measuring the convertible electrical coulombs. The measured coulombic loss in LIBs during operation is prevailingly believed as the capacity fading mainly due to parasitic reactions at the electrode/electrolyte interfaces (EEIs), including the loss of active materials (LAM) and the loss of lithium inventory (LLI) or charge inventory^11,12^. It has been the common belief that suppressed parasitic reactions with minimum coulombic loss could sustain high capacity retention for long battery lifetime. Heretofore, some specialized descriptors and protocols have been developed to probe either the quantity of parasitic coulombic loss^13,14^ or the rate of parasitic reactions^12,15^. For instance, coulombic efficiency (CE) or previously called faradaic efficiency has been adopted since the 1980s as the most common descriptor among laboratories and industries to describe the efficiency of chemical-electrical energy conversion in rechargeable batteries^16,17^. The small deviation from unity, also called coulombic inefficiency (CIE), is typical evidence of persistent parasitic reactions occurring in the cells. According to the presumably irreversible inventory loss, there have been community-wide efforts over decades in searching for battery chemistries with extremely high CE (>99.98%) so that a long battery life of 1000 charging/discharging cycles and 80% capacity retention can be assured^12^. In reality, high-performance Li-ion chemistries with long cycle life have been routinely reported from the commercialized liquid-based LIBs to the prospective solid-state lithium batteries^18–27^, while contradictorily a CE higher than 99.9% is rarely measured (Fig. 1a). Even with the help of the cutting-edge ultra-high precision charger (UHPC) equipment of 1 part per million precision (i.e., 0.0001%)^13,28^, recent censors still reveal that the CIE is far larger than the fractional capacity fading per cycle^18–20,29–31^. Herein, the parasitic coulombic loss represents a systematic overestimation to irreversible capacity fading, no matter using high precision measurements or in the optimized chemistries. To the best of current knowledge, the coulombic descriptors like CE are recommended as a semi-quantitative performance indicator for rechargeable batteries^32^.

While revisiting tremendous efforts on LIB chemistry optimizations, it is remarkably noticed that the prevailing belief of irreversible coulombic loss sometimes becomes less qualified for semi-quantitative performance evaluation. In some exceptional reports, relatively increased oxidative parasitic reactions at the positive electrode side favored performance improvement in full cells during cycling, e.g., using electrolytes additives, at higher cutoff voltage, or lower negative-electrode/positive-electrode capacity ratio^33–37^. Yet intriguingly, coulombic descriptors suggested worse CE, increased voltage-capacity slippage, increased self-discharging, or higher leakage current. The seemingly paradoxical phenomena and the systematic overestimation both imply the physical puzzles in the relationship between parasitic coulombic loss and irreversible capacity fading. Deciphering the long-standing puzzles would be fundamentally and practically essential to implement those coulombic descriptors for accurate and quantitative performance evaluation and prognostics.

The myth of macroscopic coulombic loss in full cells is decoupled from the individual roles of microscopic parasitic reactions at both electrode-electrolyte interfaces (EEIs). It has been reported that high precision leakage current (HpLC) method quantitatively measures the rate of electron transfer reactions at both EEIs, which uniquely offers an unprecedented opportunity to investigate the microscopic chemical nature of parasitic reactions^15,38–41^. To establish the relationship between parasitic coulombic loss and irreversible capacity fading, the self-discharging process in open-circuit-voltage experiments was also particularly investigated. In this work, a well-studied graphite (Gr)||LiNi_0.5_Mn_0.3_Co_0.2_O_2_ (NMC532) battery was utilized as the model chemistry. It is revealed in this work that the physical disconnection between coulombic loss and capacity fading lies in the fact that not all coulombic consumption by parasitic reactions equivalently contributes to irreversible capacity fading in full cells. This investigation uncovered two dimensionless and measurable coulombic descriptors to quantitatively connect with battery capacity in physically meaningful manners, i.e., the detrimental ratio as *ρ* and the balanced ratio as *i*_*p*_*/i*_*n*_. The versatility of these principles was further illustrated using multiple silicon (Si)-based Li-ion chemistries with various electrolytes, electrolyte additives, and positive electrodes, as well as the large-format pouch-cell demonstrations. It also implies that *ρ* and *i*_*p*_*/i*_*n*_ are two universal parameters that can be used to guide the design of high-performance batteries.

## Results

### Disconnection between coulombic measurements and reversible capacity

To elaborate the disconnection between the coulombic loss and the capacity fading without losing any generality, an investigation on Gr||NMC532 model chemistry without any modification or electrolyte additive was demonstrated for the ease of reproducibility and validation by the community (see Supplementary Fig. 1 for the basic electrochemical performance). To focus on the physics behind the coulombic measurement, Gr||NMC532 full cells were cycling at a relatively low cutoff voltage of 4.2 V to demonstrate the high-performance of the model chemistry. This model chemistry maintained about 73% of its initial reversible capacity after 1000 charging/discharging cycles (Fig. 1b), exhibiting comparable electrochemical stability with the open literature using the baseline chemistry (also summarized in Supplementary Table 1)^42–44^.

Two conventional descriptors for coulombic loss, such as CE and voltage-capacity slippage, are exemplified for the disconnected relationship with battery capacity. CE has been widely implemented as a key quality indicator among numerous rechargeable batteries. The connection between CE and capacity relies on the presumption of irreversible charge inventory loss that all coulombic loss will equivalently contribute to the capacity fading as shown in Eq. 1.

1 $${{\mbox{C}}}^{{\mbox{i}}}={{\mbox{C}}}^{0}\prod {{\mbox{CE}}}^{{\mbox{i}}}$$

where ${C}^{0}$ stands for the initial capacity, and ${C}^{i}$ and ${{CE}}^{i}$ stands for reversible capacity and coulombic efficiency at the *i*^th^ cycle, respectively. Figure 1b clearly shows that the conventional charge inventory model severely deviates from the actual performance of the cell, when both the measured CE^i^ per cycle and the reported 99.9% in the literature were used for the calculation. The apparent discrepancy underlies the overestimation of irreversible charge inventory loss towards the capacity fading in Eq. 1, rather than the random errors from inaccurate measurements and non-optimized chemistries. In addition, voltage-capacity slippage is another descriptor of coulombic consumption by parasitic reactions in the cell^45,46^. Figure 1c shows the slipping voltage profiles of Gr||NMC532 cell during charging/discharging as a function of the cumulative capacity, as calculated in Eq. 2.

2 $${{\mbox{C}}}_{{\mbox{slippage}}}=\sum ({{\mbox{C}}}^{{\mbox{i}}} * {{\mbox{CIE}}}^{{\mbox{i}}})$$

Although the voltage profile of Gr||NMC532 cell exhibits highly reversible, there is substantially large voltage-capacity slippage from accumulated coulombic consumption by parasitic reaction, also indicating the non-equivalent relationship.

Besides the discrepancy in the measurements, one should note the conceptual disconnection between them. Believing that batteries should ideally have an infinite lifespan without any parasitic reactions, it is further presumed that minimizing parasitic reactions and coulombic loss can improve capacity retention. Yet, this simple presumption systematically ignores the sophisticated impacts of parasitic reactions, leading to weak guarantees for capacity improvements. For instance, it was reported that LiO-t-C_4_F_9_ electrolyte additive can improve capacity retention in full cells, associated with increased voltage-capacity slippage^33^. It is a sacrifice-type additive for preferential decomposition to form a stable interface on the surface of positive electrode, but its decomposition causes additional coulombic consumption. Therefore, we mainly argue in this study that coulombic loss is physically disconnected from capacity fading. Probing the physics behind coulombic loss is the key to connecting with battery performance.

### Correlation between parasitic coulombic loss and capacity fading

**Fig. 2:**
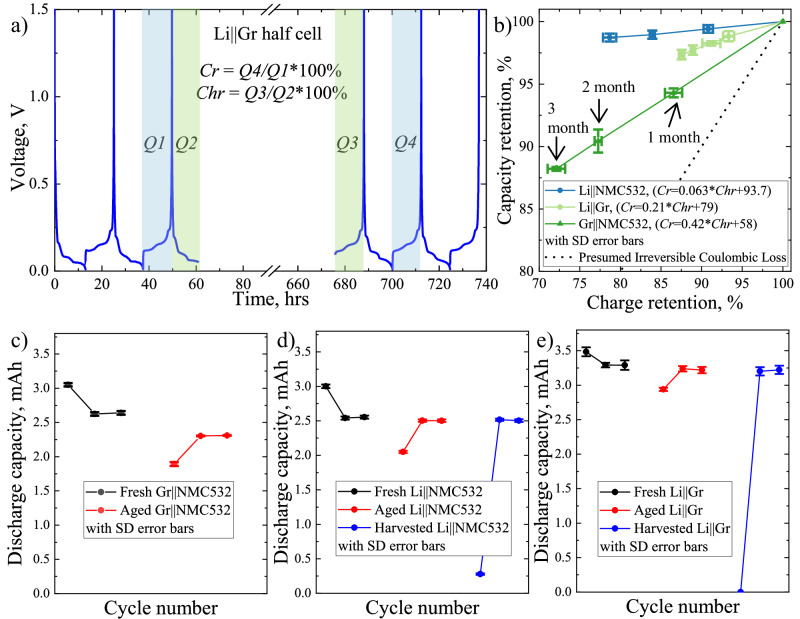
OCV-RPT showcasing identical aging behavior of electrodes in both half cells and full cells. **a** Typical voltage profiles of a Li||Gr cell before and after OCV aging for 600 h. **b** Linear relationship between the capacity retention and the charge retention for Li||NMC532, Li||Gr, and Gr||NMC532 cells. A reference for the prevailingly believed irreversible coulombic loss with a slope of 1 is also provided in the black dash line, showing apparent deviation from the realistic situation. Comparison of discharge capacity at C/10 for (**c**) Gr||NMC532, (**d**) Li||NMC532, and (**e**) Li||Gr at various stages (before OCV, after 3-month OCV, and harvested half cells after OCV of a full cell). The current rate follow 1C_Gr||NMC532_ = 2.72 mA, 1C_Li||NMC532_ = 2.72 mA, 1C_Li||Gr_ = 3.68 mA. The measured capacity shows the mean value of three repeating cells in the center with SD error bars.

Unsurprisingly in the data presented above, the quantitative relationship between the coulombic loss and the capacity retention was overwhelmed by the overestimation. To investigate the physical gap, aging tests at open-circuit voltage (OCV) were adopted to strategically amplify the coulombic loss and capacity fading. The typical procedure of OCV aging experiment is shown in Fig. 2a. The cells were fully charged to 100% state-of-charge (SOC) (i.e., 0.05 V for Li||Gr, 4.3 V for Li||NMC532, and 4.25 V for Gr||NMC532) after 3 formation cycles with a constant current of C/10 (1C_Li||Gr_ = 3.68 mA, 1C_Li||NMC532_ = 2.72 mA, 1C_Gr||NMC532_ = 2.72 mA) and then stored at 30 °C for continuous OCV aging up to 3 months. After the OCV aging, a reference performance test (RPT) was conducted by cycling the cells at C/10 for 3 cycles. The Gr||NMC532 full cells after OCV-RPT tests for 3 months were discharged to 2.5 V and then dissembled to harvest NMC532 and Gr electrodes and prepare them in half cells using fresh electrolyte and Li metal counter electrode. The harvested NMC532 in half cells were first discharged to 0% SOC at 2.5 V vs Li/Li^+^ at C/10 current rate, showing a 0.278 mAh discharging capacity (about 11% of initial capacity) due to lithium vacancy in the positive electrode (Fig. 2d). In comparison, the harvested Gr in half cells had no discharging/delithiation capacity to 0% SOC at 1.5 V vs Li/Li^+^ (Fig. 2e). This 11% capacity loss could represent the loss of lithium inventory during their full-cell operation. Subsequently, both harvested positive and negative electrodes in half cells were cycled at C/10 for two cycles to show 2.503 mAh and 3.222 mAh discharge capacity, respectively. It is found that the electrochemical performance of harvested NMC532 and Gr from full cells is almost identical to those 3-month aged in half-cell configurations, 2.503 mAh in Li | |NMC532 cells and 3.220 mAh in Li||Gr cells (Fig. 2c–e and Supplementary Fig. 2). This confirms that the aging behavior of electrode materials in half cells is well representative to those aged in full cells, indicating minor crosstalk influence at a relatively low cutoff voltage of 4.3 V. To avoid confusion, the charge process is referred to the removal of Li from the positive electrode and/or the insertion of Li into the negative electrode, and vice versa.

The coulombic loss (*1-Chr*) during OCV aging is evaluated by the charge retention (*Chr*) due to the self-discharging behavior, which is defined as the ratio between Q3 (the first discharge capacity of RPT) and Q2 (the last charge capacity before OCV aging) as Eq. 3.

3 $${\mbox{Chr}}=\frac{{\mbox{Q}}3}{{\mbox{Q}}2} * 100\%$$

The capacity fading (*1-Cr*) during OCV aging is evaluated by the cycling capacity retention (*Cr*), which is defined as the ratio between Q4 (i.e., the 2^nd^ discharge capacity in RPT) and Q1 (i.e., the 3^rd^ discharge capacity before OCV aging) as Eq. 4.

4 $${\mbox{Cr}}=\frac{{\mbox{Q}}4}{{\mbox{Q}}1} * 100\%$$

**Fig. 3:**
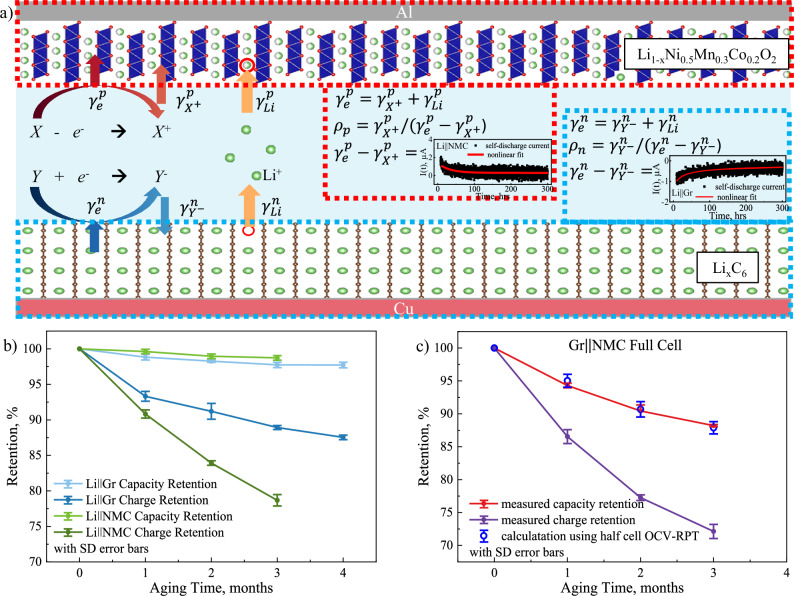
Charge balance at electrode/electrolyte interfaces to achieve local charge neutrality. **a** Schematic diagram of local charge neutrality and charge-to-capacity relationship at both electrode/electrolyte interfaces in a model chemistry of Gr||NMC532 cells at the charged state. The symbols of X and Y stand for the electrolyte species involved in the electrochemical oxidation and reduction reactions at the interface, with the byproducts of X^+^ and Y^−^, respectively. The red dash box attached on the NMC532 positive electrode illustrates the relationship between several charge transfer mechanisms at CEI for local charge neutrality, and the inlet figure is HpLC measurement of net charge transfer across CEI. Similarly, the blue dash box on Gr negative electrode shows the local charge neutrality at SEI, and the inlet figure is net charge transfer across SEI via HpLC. **b** OCV-RPT tests of Li||NMC532 half cells up to 3 months and Li||Gr half cells up to 4 months at their charge state of 4.3 V and 0.05 V, respectively. **c** Good agreement between the analytical model in Eq. 9 using half-cell data and the experimental measurements in full cells. The measured retention data shows the mean value of three repeating cells in the center with SD error bars.

The relationship between the measured charge retention (*Chr*) and capacity retention (*Cr*) for different cell chemistries is shown in Fig. 2b (see Supplementary Fig. 2 for the voltage profiles of all cells before and after OCV aging, and Fig. 3b, c for measured *Chr* and *Cr* as functions of the aging time). Remarkably, a strongly linear relationship between charge retention and capacity retention was observed for all three types of cells (Li||Gr, Li||NMC532, and Gr||NMC532). However, their linear slopes of less than 1 show apparent deviation from the presumed irreversible coulombic loss in Eq. 1, suggesting that only parts of parasitic coulombic consumption are responsible for capacity fading.

This phenomenon is conceptually supported by many individual LIB chemistries. At the surface of graphite negative electrode, electrolyte solvents decomposed reductively to deposit organic lithium salt products (e.g., lithium alkyl carbonates) as a passivating solid-electrolyte interphase (SEI)^11,47^. According to Besenhard model and numerous physical observations, partial organic byproducts in SEI also co-intercalate into graphite sheets at the edge sites when the interlayer distance of graphite expands during Li intercalation^47^. Depending on the electrolyte compositions of carbonate- or ether-based solvents, the co-intercalated organic byproducts more or less penetrate into the bulk structure of graphite during cell operation and become electrochemically inactive for deintercalation, resulting in exfoliation and electrical isolation of graphite fragments for irreversible capacity fading^11,48,49^. Likewise, at the surface of metal-oxide positive electrodes, electrochemical oxidation of electrolyte species (e.g., ethylene carbonate or EC) becomes organic radical cations (e.g., EC^+^) and further decomposes to solid byproducts (e.g., polycarbonates) depositing on a cathode/electrolyte interphase (CEI)^11^. Meanwhile, the organic radical cations also proceed deprotonation reactions to charge-neutralize the organic framework by releasing protons (H^+^) or acidic organic fragments near the CEI, resulting in a locally highly acidic environment^41,50^. The incorporation of acidic protons or proton-bearing species chronically affects the structural integrity of metal-oxide electrode materials for irreversible capacity fading, such as chemically acidic corrosion of transition metals and oxygen in the lattice, and recently unearthed chemical-modulated structural degradation of irreversible layered to rock-salt phase transformation and solvated structure with limited Li active sites^41,50,51^. Moreover, electrolyte additives are particularly designed for preferential decomposition to form a stable EEI and suppress those detrimental factors in the original parasitic reactions, probably at the burden of increased coulombic consumption^11,33^. For instance, 3-hexylthiophene (3HT) additive is preferentially oxidized during charging to form a corrosion-inhibitor film at the surface of NMC622 positive electrode that prevents the severe attack from acidic proton-bearing species and improves capacity retention of Li||NMC622 cells at 4.5 V^40^. Yet, the preferential decomposition of 3HT additive increases the rate of parasitic reactions with more burden of coulombic consumption. Herein, one should realize the dual effects of the parasitic reactions at EEIs, including direct coulombic consumption as the burden of charge inventory and partial generation of detrimental byproducts for loss of active material. As exhibited by the slope of less than 1 in Fig. 2b, the coulombic loss of parasitic reactions partially contributes to the irreversible capacity fading. Now, note that the failure of Eq. 1 lies on the implicit assumption that all parasitic coulombic consumption is equivalent to irreversible capacity fading.

The physical meaning of the slope is schematically presented in Fig. 3a for summarizing several classes of possible charge transfer reactions across the EEIs. It is well accepted that some electrolyte components, such as solvent molecules, salt anions and additives, can be slowly reduced at the surface of negative electrode to form SEI and oxidized at the positive electrode surface to generate CEI, resulting in a loss of electrons (${\gamma }_{e}^{n}$) in the negative electrode and a gain of electrons in the positive electrode $({\gamma }_{e}^{p})$, respectively. Thus, ${\gamma }_{e}^{n}$ and ${\gamma }_{e}^{p}$ are thermodynamic terms that describe the rate of electron transfer in electrolyte decompositions at the electrode/electrolyte interfaces, regardless of the external measurements. Meanwhile, electrolyte oxidation or reduction generates positively or negatively charged species and radicals, *X*^*+*^ or *Y*^*-*^, respectively. A fraction of the generated *X*^*+*^ or *Y*^*-*^ diffuse away from the electrode and into the bulk electrolyte, usually defined as crosstalk. The rest byproducts (${\gamma }_{{X}^{+}}^{p}$ or ${\gamma }_{{Y}^{-}}^{n}$) are deposited on electrode surface for detrimental contributions to permanent capacity loss, like aforementioned detrimental proton-bearing (H^+^) species from electrolyte oxidation^41,50,51^. Therefore, the deposited charged species (e.g., ${\gamma }_{{X}^{+}}^{p}$) is always a fraction of the charge consumption of electrolyte decomposition (e.g., ${\gamma }_{e}^{p}$). The difference between ${\gamma }_{e}^{p}$ and ${\gamma }_{{X}^{+}}^{p}$ can describe the contribution of crosstalk, so that the capacity retention of full cells can be roughly calculated using data from half cells. Using the positive electrode boxed in red dotted rectangle as an isolated system, the local charge neutrality must be maintained to accommodate all charge transfer mechanisms. To satisfy the local charge neutrality during OCV aging, ${\gamma }_{{Li}}^{p}$ is a charge balancing mechanism by self-discharging process of the positive electrode, accounting for the byproducts diffusing into the bulk electrolyte, as Eq. 5.

5 $${{{\rm{\gamma }}}}_{{\mbox{e}}}^{{\mbox{p}}}={{{\rm{\gamma }}}}_{{{\mbox{X}}}^{+}}^{{\mbox{p}}}+{{{\rm{\gamma }}}}_{{\mbox{Li}}}^{{\mbox{p}}}$$

In OCV-RPT tests, the net of electron transfer across the CEI (${\gamma }_{e}^{p}-{\gamma }_{{X}^{+}}^{p}$) equivalently contributes to the charge loss (*1-Chr*) due to self-discharge process (${\gamma }_{{Li}}^{p}$). Therefore, the byproducts from electrolyte oxidation $({\gamma }_{{X}^{+}}^{p})$ partially contribute to the permanent loss of reversible capacity (*1-Cr*), which is a fraction of the net electron transfer (${\gamma }_{e}^{p}-{\gamma }_{{X}^{+}}^{p}$), as shown in Eq. 6.

6 $${{{\rm{\rho }}}}_{{\mbox{p}}}={{{\rm{\gamma }}}}_{{{\mbox{X}}}^{+}}^{{\mbox{p}}}/({{{\rm{\gamma }}}}_{{\mbox{e}}}^{{\mbox{p}}}{-{{\rm{\gamma }}}}_{{{\mbox{X}}}^{+}}^{{\mbox{p}}})$$

It is also worth emphasizing that, when cells are at constant-voltage charging mode in HpLC measurements, the rate of Li back insertion (${\gamma }_{{Li}}^{p}$) will approach to zero due to no self-discharging, and the net of electron transfer (${\gamma }_{e}^{p}-{\gamma }_{{X}^{+}}^{p}$) will be compensated by the counter Li metal electrode through the external circuit and measured as the leakage current.

The same charge balance mechanism is also preserved at the SEI for the negative electrode, as shown in Fig. 3a, and the detrimental factors (${\gamma }_{{Y}^{-}}^{n}$) are some chemistry-dependent byproducts in parasitic reactions at the surface of negative electrode^11,48,49^. Nonetheless, this definition of slope in Eq. 6 is conditionally true in half cells, where a thick metallic Li electrode has no Li inventory limit for Li stripping, and the self-discharge portion can only reduce the capacity on the immediately following discharge process (Q3 in Fig. 2a) and have no permanent impact on the reversible capacity (Q4 in Fig. 2a). When it comes to the inventory limited scenario in full cells, another mechanism of inventory compensation kicks in.

### Charge inventory compensation between the positive electrode and negative electrode

What is surprising is that the slopes between the capacity retention and the charge retention are quite different from each cell configuration (as shown in Fig. 2b, ${\rho }_{f}$ for Gr||NMC532 full cells, ${\rho }_{n}$ for Li||Gr half cells, and ${\rho }_{p}$ for Li||NMC532 half cells). This arises from another puzzle surrounding the impacts of parasitic coulombic consumption (e.g., ${\gamma }_{e}^{p}-{\gamma }_{{X}^{+}}^{p}$) at individual electrodes on the global charge inventory within the full cells. Insufficient comprehension creates confusion over paradoxical phenomenon wherein full cells exhibited the improved capacity despite increased parasitic coulombic consumption on the positive electrode side^33–35^. This confusion also impedes the development of novel battery chemistries.

It was prevailingly presumed that coulombic consumption by parasitic reactions causes the irreversible loss of charge inventory, which has been widely applied in many coulombic measurements for performance evaluation. However, this is also a misconception that disconnects coulombic loss from the capacity loss in full cells, because it systematically ignores the distinct manners of coulombic consumption between positive electrode and negative electrode. One should note that the net electrons released from oxidative parasitic reactions at CEI $({\gamma }_{e}^{p}-{\gamma }_{{X}^{+}}^{p})$ causes the Li inventory gains in the positive electrode (${\gamma }_{{Li}}^{p}$) in the self-discharge process for maintaining local charge neutrally, while the net electrons consumed by reductive parasitic reactions at SEI (${\gamma }_{e}^{n}-{\gamma }_{{Y}^{-}}^{n}$) causes the Li inventory vacancies in the negative electrode (${\gamma }_{{Li}}^{n}$). Due to opposite inventory changes between positive electrode and negative electrode, it strongly argues that the coulombic consumption in full cells follows the irreversible charge inventory loss in the prevailing belief. In reality, the net change of charge inventory in full cells should involve a global charge inventory compensation mechanism that supplies from ${\gamma }_{{Li}}^{p}$ to compensate ${\gamma }_{{Li}}^{n}$ to sustain certain reversible capacity. The compensated inventory depends on balancing coulombic consumption between both electrodes, which is equal to the minimum accessible charge inventory of either the Li supply in the positive electrode or the Li vacancy in the negative electrode, in Eq. 7.

7 $${{\rm{Compensated}}}\; {{\rm{charge}}}\; {{\rm{inventory}}}=\min ({{{\rm{\gamma }}}}_{{\mbox{Li}}}^{{\mbox{p}}},{{{\rm{\gamma }}}}_{{\mbox{Li}}}^{{\mbox{n}}})$$

Theoretically, the usable inventory in full cells can remain unchanged and will not reduce reversible capacity, as long as balancing coulombic consumption between positive electrode and negative electrode, or having infinite inventory like excessive Li metal electrode. Similar findings have been previously reported that improving the current efficiency of positive electrode and negative electrode can increase battery capacity retention^52,53^.

**Fig. 4:**
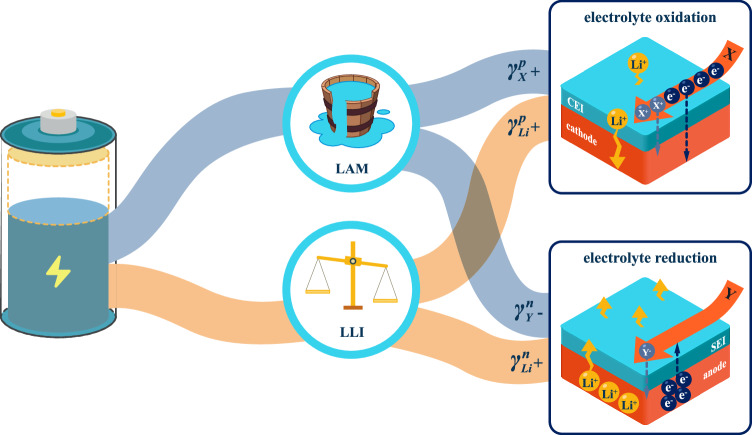
The physical connection of parasitic coulombic loss at individual electrodes and the remaining capacity in LIB full cells, underlying the synergistic effects of local charge neutrality and global charge inventory compensation.

In this study, it has been noticed that the coulombic consumption model of both global inventory compensation, $\min ({\gamma }_{{Li}}^{p},{\gamma }_{{Li}}^{c})$, and the slope ratio between capacity loss and coulombic consumption, *ρ*, play the synergistic effects on the reversible capacity in LIBs (as illustrated in Fig. 4). The capacity loss in the full cells can be physically connected to coulombic consumption due to parasitic reactions in Eq. 8.

8 $${{\rm{Capacity}}} \, {{\rm{loss}}}=\max \left({{{\rm{\gamma }}}}_{{{\mbox{X}}}^{+}}^{{\mbox{p}}},{{{\rm{\gamma }}}}_{{{\mbox{Y}}}^{-}}^{{\mbox{n}}}\right)+{\mbox{|}}{{{\rm{\gamma }}}}_{{\mbox{Li}}}^{{\mbox{p}}}-{{{\rm{\gamma }}}}_{{\mbox{Li}}}^{{\mbox{n}}}{\mbox{|}}$$

where ${\gamma }_{{X}^{+}}^{p}$ and ${\gamma }_{{Y}^{-}}^{n}$ are the loss of irreversible capacity in individual electrodes and $|{\gamma }_{{Li}}^{p}-{\gamma }_{{Li}}^{n}|$ stands for the loss of uncompensated charge inventory due to imbalanced charge consumptions between both electrodes. Therefore, a mathematical approach can be established for the capacity retention of the full cells (see Supplementary Note 1), including charge consumption by the parasitic reactions and the global charge inventory compensation between both electrodes, as shown in Eq. 9.

9 $${{\mbox{Cr}}}_{{\mbox{f}}}=	\min \left({{\mbox{Chr}}}_{n},{{\mbox{Chr}}}_{{\mbox{p}}}\right)+\min \Big((1-{{{\rm{\rho }}}}_{{\mbox{p}}}) * \left(1-{{\mbox{Chr}}}_{{\mbox{p}}}\right),(1-{{{\rm{\rho }}}}_{{\mbox{n}}}) \\ 	* \left(1-{{\mbox{Chr}}}_{{\mbox{n}}}\right)\Big)$$

In above equation, ${{Cr}}_{f}$ is the capacity retention of the full cell after aging, ${{Chr}}_{n}$ and ${{Chr}}_{p}$ are the charge retention of negative electrode and positive electrode, respectively, for the inventory compensation term, ${\rho }_{n}$ and ${\rho }_{p}$ are the detrimental ratios between the charge loss from parasitic reactions to the capacity loss in the negative electrode and the positive electrode, respectively. Figure 3b shows the evolution of the charge retention (*Chr*) and capacity reduction (*Cr*) during OCV aging for both Li | |Gr and Li | |NMC532 half cells. Using the data shown in Fig. 3b, Eq. 9 can mathematically reproduce the capacity retention of Gr | |NMC532 full cells during OCV aging (see Fig. 3c). Note that the failure of irreversible charge loss model in Eq. 1 systematically ignores the second term of global charge inventory compensation mechanism and inaccurately describes charge-capacity relationship without the partial correlation (i.e., ${\rho }_{n}$ and ${\rho }_{p}$) in Eq. 9. This clearly demonstrates that these two terms are the missing puzzles to physically connect the coulombic consumption to the capacity retention.

### Analytically reconstructing the puzzle for rapid performance prognostics

**Fig. 5:**
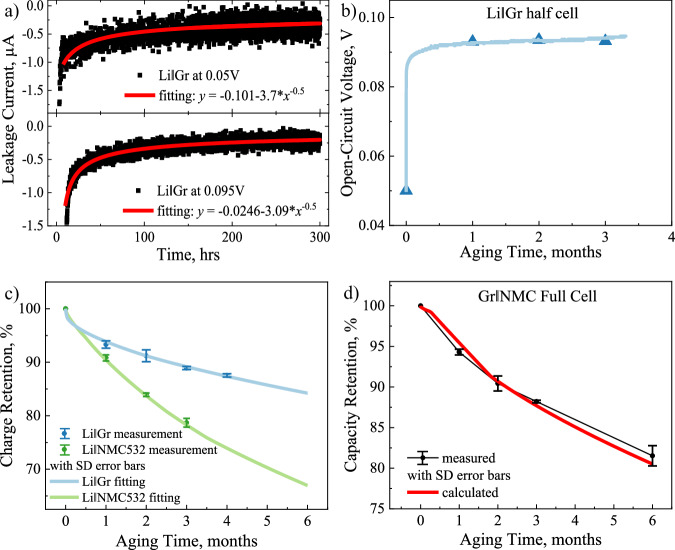
Development of analytical solutions to rapid prediction of battery life based on the hypothetical model. **a** HpLC measurements of Li||Gr at 0.05 V and 0.095 V, with the power-law decay fitting to measure the steady leakage current values at different state of charge. **b** The open-circuit voltage evolution of Li||Gr half cells during calendar aging, initiating at the charge state of 0.05 V. The blue triangle datapoints are the discrete measurements after OCV aging without wire connection, which show consistent results with the continuous measurements during OCV aging with wire connection in the light-blue line. **c** Estimation of the calculated charge retention via HpLC measurements for 300 h to the OCV-RPT measured charge retention up to 4 months in both Li||NMC532 and Li||Gr half cells. **d** Reliable prediction of OCV-RPT full cell data up to 6 months by the numerical approach of using accelerated HpLC testing protocol in half cell in 300 h (0.5 months), derived from the analytical approach of the calculated OCV-RPT half-cell data. The measured retention data shows the mean value of three repeating cells in the center with SD error bars.

Effort will be made here to draw an analytical connection between the coulombic loss and the cell performance and demonstrate its potential capability of rapid performance prognostics. HpLC measurement is a novel technique to quantitatively track the rate of electron transfer reactions at the heterogeneous interfaces, such as CEI and SEI, and the measured leakage current profiles indicate the time-evolving kinetics of parasitic reactions^15,38^. Figure 5a shows a set of typical data collected during leakage current measurement. The cell, preferably a half cell with Li metal electrode, was constant voltage charged to a specific potential for the extended time. Thus, the electron transfer cross the interface (e.g., ${\gamma }_{e}^{p}-{\gamma }_{{X}^{+}}^{p}$ at CEI) cannot be compensated by self-discharging of electrode materials (e.g., ${\gamma }_{{Li}}^{p}$), instead passing through the external circuit to Li-metal counter electrode as recorded in leakage current profiles (see Fig. 5a). The leakage current rapidly decays at the initial experimental time for equilibrating Li^+^ concentration gradient in the cells, but subsequentially stabilizes to non-zero current values even after formation cycles and extensive aging time like 300 h in Fig. 5a. Therefore, the leakage current during voltage holding represents the continuous electron transfer across the interfaces because of the persistent parasitic reactions. Two possible SEI growth models (Eq. 10 and 11) are investigated here to mathematically describe the time-evolution of leakage current or parasitic reactions.

10 $${{\rm{Void}}}-{{\rm{filling\; model}}}:{{\rm{i}}}({{\rm{t}}})={{\mbox{i}}}_{0}{{\mbox{e}}}^{-{\mbox{at}}}+{{\mbox{i}}}_{\infty }$$

11 $${{\rm{Continuous}}}-{{\rm{growth\; model}}}:{{\rm{i}}}({{\rm{t}}})=\frac{{{\mbox{i}}}_{0}}{\sqrt{{\mbox{t}}-{{\mbox{t}}}_{0}}}+{{\mbox{i}}}_{\infty }$$

The void-filling model tends to overestimate the steady leakage current, while the continuous-growth model tends to underestimate the steady leakage current (see Supplementary Fig. 3). This discrepancy hints that the actual SEI growth model can include both the void-filling component, healing of damaged SEI, and the continuous-growth component, thickening of SEI. It is reasonably assumed here that the continuous growth model plays a dominant role in calendar-type testing like OCV aging and voltage-holding testing, and that the void filling can play a bigger role in dynamic testing like repeating charge/discharge cycling.

In principle, the integration of *i(t)* represents the coulombic loss from parasitic reaction during an aging period of *t*. Besides, the rate of parasitic reactions is also dependent of the working potential at the interfaces, following electrochemical characteristics as Tafel equation. For example, Li||Gr cells in OCV aging tests show the relaxation of open circuit potential as a function of the aging time (Fig. 5b and other chemistries in Supplementary Fig. 4). The cells were charged to 0.05 V before OCV aging and promptly relaxed to about 0.095 V, because of accompanying removal of Li from Li_x_C_6_ to compensate the coulombic consumption of reductive parasitic reactions. The calculations of individual leakage current profiles at 0.05 or 0.095 V (Fig. 5a) present the underestimation or overestimation to the measured charge retention during OCV aging (as shown in Supplementary Fig. 5), respectively. Herein, taking the varying OCV potential into account, charge retention can be mathematically described in Eq. 12.

12 $${\mbox{Chr}}\left({\mbox{t}}\right)=1-{\int }_{0}^{{\mbox{t}}}{\mbox{i}}\left({\mbox{t}},{\mbox{V}}({\mbox{t}})\right){\mbox{dt}}$$

A compromised approach is demonstrated to calculate the charge retention in the negative electrode using both sets of 0.05 V and 0.095 V data, as shown in Eq. 13.

13 $${{\mbox{Chr}}}_{{\mbox{n}}}\left({\mbox{t}}\right)=1-{\int }_{0}^{40}{{\mbox{i}}}_{{\mbox{n}}}\left({\mbox{t,}}0.05{\mbox{V}}\right){\mbox{dt}}-{\int }_{40}^{{\mbox{t}}}{{\mbox{i}}}_{{\mbox{n}}}\left({\mbox{t,}}0.095{\mbox{V}}\right){\mbox{dt}}$$

where 40 h approximate the initial time of prompt voltage evolution (Fig. 5b) for a prototyping demonstration. Figure 5c clearly shows that the charge retention of Li||Gr cell during long-term OCV aging (around 3000 h) can be successfully calculated by using the HpLC experiment (up to 300 h). The same numerical manipulation was adopted to calculate the charge retention of Li||NMC532 cell using Eq. 14 with great agreement with the experimental values (see Fig. 5c and HpLC data and voltage-time relaxation in Supplementary Fig. 6).

14 $${{\mbox{Chr}}}_{{\mbox{p}}}\left({\mbox{t}}\right)=1-{\int }_{0}^{1200}{{\mbox{i}}}_{{\mbox{p}}}\left({\mbox{t,}}4.3{\mbox{V}}\right){\mbox{dt}}-{\int }_{1200}^{{\mbox{t}}}{{\mbox{i}}}_{{\mbox{p}}}\left({\mbox{t,}}4.04{\mbox{V}}\right){\mbox{dt}}$$

One can further substitute the calculated capacity retention of individual electrode half cells from Eqs. 13 and 14 into Eq. 9 to numerically calculate the capacity retention of Gr||NMC532 full cells. Given the ability of 300-h or 0.5-month short-term HpLC measurements, an analytical solution that relies on the accurate physical description of coulombic loss exhibits a good agreement to the measured capacity retention up to 6 months (Fig. 5d).

## Discussion

**Fig. 6:**
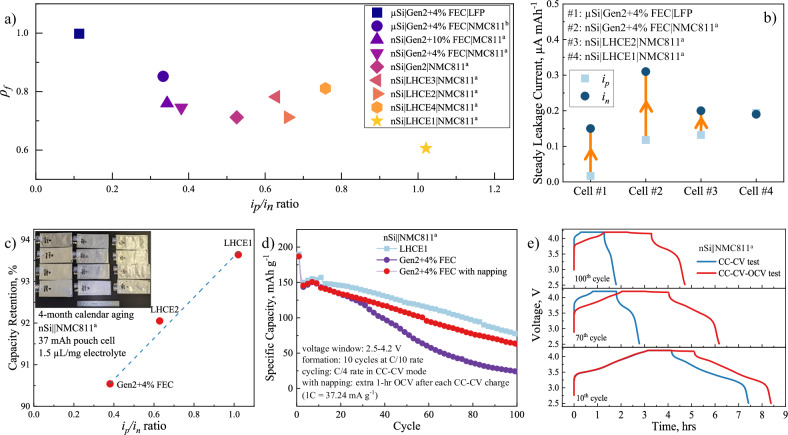
Implementing the hypothetical coulombic consumption mechanism with measurable parameters in Si-based Li-ion chemistries. **a** Strong correlation of charge balance parameter (*i*_*p*_*/i*_*n*_) via HpLC tests and the full cell performance indicator (*ρ*_*f*_) via OCV-RPT tests, revealing the importance of charge inventory compensation mechanisms. **b** The ratio of steady leakage current between the positive and negative electrode is dependent of cell chemistries. The red arrow suggests the inventory deficit from more coulombic consumption at the negative electrode. The experimental validations of the hypothetical coulombic consumption mechanisms via (**c**) calendar aging tests and (**d**) cycling tests of nSi||NMC811^a^ cell performance using large-format pouch cells. **e** The conceptual demonstration of having global charge inventory compensation to improve stable cycling performance by incorporating additional napping OCV steps in charge/discharge cycling after 10 formation cycles.

The above study on the Gr||NMC532 model chemistry reveals two important dimensionless parameters for coulombic loss in the physical manners, *ρ* and *i*_*p*_*/i*_*n*_, that can be easily measured and used to guide the chemistry development for long-life batteries. In principle, a small *ρ* in full cells, ideally <0.2, is highly desired for long calendar batteries, because numerically the intercept of the straight line at *Chr* = 0 is the ultimate capacity retention of the cell, given that the parasitic reactions consume all the stored charge. Meanwhile, a balanced *i*_*p*_*/i*_*n*_ ratio, representing the charge compensation between the positive electrode $({\gamma }_{{Li}}^{p})$ and the negative electrode $({\gamma }_{{Li}}^{n})$, can be used as a semi-empirical criterion to minimize the cyclable charge inventory loss with an anticipated ratio of 1. The following is a case study using Si-based negative electrode to showcase how these two dimensionless parameters can be adopted for battery development. This case study covers 9 different Li-ion chemistries using 2 positive electrodes, 2 Si-based negative electrodes, 5 electrolytes, and 1 electrolyte additive (*i*_*p*_*/i*_*n*_ via HpLC measurements in Supplementary Fig. 7, *ρ*_*f*_ via full-cell OCV-RPT tests in Supplementary Figs. 8–9, and outlined in Supplementary Table 2). Figure 6a shows the correlation between the measured *ρ*_*f*_ of full cells during OCV aging and the measured *i*_*p*_*/i*_*n*_ via HpLC measurement of half cells for multiple chemistries. It is clearly shown that the performance indicator *ρ*_*f*_ for full cells is strongly dependent on the ratio of electron transfer reactions between positive and negative electrodes (*i*_*p*_*/i*_*n*_). The value of *ρ*_*f*_ steadily declines as *i*_*p*_*/i*_*n*_ increases up to 1.0.

In the extreme case when micro-size Si (μSi) was paired with LiFePO_4_ (LFP) and 1.2 M LiPF_6_ in ethylene carbonate/ethyl methyl carbonate (EC/EMC, 3:7 by weight), denoted as Gen2, with 4 wt% fluorinated ethylene carbonate (FEC) was used as the electrolyte, the cell showed a poor capacity retention during the OCV aging, losing 38% reversible capacity within 3 months. The poor performance can be rooted to the extremely low interfacial reactivity of FePO_4_ at the resting potential (Fig. 6b); very limited bonus Li inventory was generated during the OCV aging. Therefore, all coulombic loss at the negative electrode equivalently contributes to the capacity loss of the full cell with *ρ*_*f*_ = 1. When a more reactive positive electrode NMC811 was used to pair with nano-size Si (nSi), the measured steady leakage current for both the negative electrode and positive electrode increased significantly (see Fig. 6b), but the ratio increased to 0.33, leading to a slight decrease of *ρ*_*f*_ value for full cells to about 0.85. Aligned with the expectation, a slightly better capacity retention was observed (~10% capacity loss within 4-month OCV aging in Fig. 6c). By replacing the carbonate-based electrolyte with a localized high-concentration electrolyte of LiFSI in tetramethylenesulfone (TMS) and 1,1,2,2-tetrafluoroethyl 2,2,3,3-tetrafluoropropylether (TTE) at 1:3:3 by mol., denoted as LHCE2, the steady leakage current on the negative electrode reduced, and that on the positive electrode increased, resulted in a higher *i*_*p*_*/i*_*n*_ (0.66), and a lower *ρ*_*f*_ (0.71), as well as a reduced capacity loss (~8% in 4 months) for full cells. The best electrochemical performance of pouch cells (Fig. 6c and voltage profiles in Supplementary Fig. 10) was observed for the nSi||NMC811^a^ cell chemistry using LHCE1 (LiFSI-DMC-TTE at 1:2.2:3 by mol., where DMC stands for dimethyl carbonate) as the electrolyte when *i*_*p*_*/i*_*n*_ approached 1.0. Figure 6c exhibits the strong dependence of balanced charge consumptions (*i*_*p*_*/i*_*n*_ ratio) on the capacity retention of Si-based lithium-ion batteries during calendar aging. The improvements in calendar life benefit from the global charge inventory compensation of balanced *i*_*p*_*/i*_*n*_, reducing the loss of lithium inventory (Supplementary Fig. 11).

This global compensation mechanism also plays a more important role in cycling life. Because of voltage manipulation of both electrodes in the cycling tests, the electrons gained from the oxidative parasitic reactions at the positive electrode (${\gamma }_{e}^{p}-{\gamma }_{{X}^{+}}^{p}$) are drained by the negative electrode through the external electrical circuit to charge-neutralize the required electrons for the reductive parasitic reactions (${\gamma }_{e}^{n}-{\gamma }_{{Y}^{-}}^{n}$). This leads to no self-discharge process in the positive electrode to resupply lithium inventory for global compensation, which aggravates capacity fading in full cells. For some positive electrodes using 80% SOC, like NMC811 at 4.2 V, full cells undergo the undesired voltage slippage during cycling to extract more Li inventory in the deeply delithiated positive electrode at higher SOC. However, the Li inventory at high SOC is limited to accommodate the continuous loss of charge inventory as cycling, again resulting in aggravated capacity fading after certain cycles (see nSi|Gen2 + 4% FEC|NMC811^a^ in Fig. 6d), as well as other consequences of positive electrode’s structure degradation and electrolyte depletion at higher voltage. In order to examine the global charge compensation and discard other possible influences using different electrolytes, a napping procedure of OCV step was intentionally incorporated at the end of charging process after 10 formation cycles to permit certain self-discharging or re-lithiation at the positive electrode. Compared with regular charging/discharging procedure (see voltage profiles of formation cycles in Supplementary Fig. 12), the same cell chemistry (nSi|Gen2 + 4% FEC|NMC811^a^) takes proper napping of self-discharging in the positive electrode to recover Li inventory and exhibits significant improvement in full cell performance (Fig. 6d, e). This reveals that global charge inventory compensation is favorable for sustaining stable performance from the continuous loss of Li inventory. It should be cautiously noted that, nevertheless, the napping procedure intentionally implemented for conceptual proving on global charge compensation mechanisms might not be suitable for practical applications, due to aggravated electrolyte depletion and electrode degradation for an extended time at high voltage. Certainly, the wisest choice is balancing the charge consumptions between the positive electrode and the negative electrode to minimize the loss of Li inventory and improve cycling performance, as shown in the optimal performance using the balanced *i*_*p*_*/i*_*n*_ LHCE1 electrolyte (Fig. 6d). Both the *i*_*p*_*/i*_*n*_-optimized chemistry and napping procedure have experimentally validated that the global charge inventory compensation underlies the self-regulation mechanism to suppress the loss of lithium inventory and improve battery life. The state-of-the-art prelithiation has been widely employed as a compromised mitigation strategy to initially supply extra lithium inventory to the negative electrode during manufacturing for accommodating lithium loss during cycling. Despite improved capacity from the initial cycles, the full cells still suffer a similar trend of capacity fading to no prelithiated full cells, because of the ignorance of the continuous lithium inventory loss from imbalanced charge consumption. Therefore, primary attention should be paid to the balanced charge consumption to enable the intrinsically global charge inventory compensation for bringing down the rate of lithium inventory loss.

Although Fig. 6b shows that the gap between *i*_*p*_ and *i*_*n*_ (*i*_*n*_ > *i*_*p*_) can be closed by increasing the value for *i*_*p*_ to improve the capacity retention of full cells, this strategy should be cautiously implemented. In a lean electrolyte system or commercialized cells, the increased leakage current can be translated into an accelerated consumption of the electrolyte components, following with an early depletion of the electrolyte in the cell. This can trigger alternative failure mechanism that is not covered in this work. For the sake of the battery design, reducing *i*_*n*_ would be a practical way to improve battery performance. Nonetheless, the performance of the best Si-based cells using LHCE1 (as seen in Fig. 6c) is still far from being satisfactory mostly due to their high *ρ*_*f*_ value (~0.6), which is higher than that of Gr||NMC532 chemistry (0.42), as well as the ideal value of 0.2. More efforts on novel electrolyte technology or Si modification to bring down *ρ*_*f*_ are highly desired to enable Si-based long-life Li-ion chemistry^6^. Furthermore, this model captures the majority of charge transfer mechanisms inside a cell. There are still many important contributors for battery performance to be included, such as (1) the fraction of crosstalk species that accept/donate electrons from/to the counter electrode and/or deposited on the surface of the counter electrode, (2) the engineering procedures of the overhang of negative electrode, N/P ratio, and electrolyte/electrode ratio, and (3) other aspects like cell operation modes in real fields. These contributors can be investigated using two recommended dimensionless criteria (*i*_*p*_*/i*_*n*_ and *ρ*) in this study to establish their physical descriptors to battery life. It would be vital to establish sufficient dimension and modality of charge consumption mechanism for accurate prediction of various battery chemistries and conditions.

We have demonstrated, through rigorous electrochemical characterizations and high precision measurements for electron transfer across the electrode/electrolyte interface, that not all coulombic consumptions by parasitic reactions are irreversible to battery capacity, against the prevailingly believed irreversible coulombic loss. This study experimentally and conceptually elaborated that the physical disconnection between coulombic loss and battery life originated from ignorance of local charge neutrality and global charge inventory compensation mechanisms in the cells. On the one hand, at local electrode/electrolyte interface, partial byproducts in parasitic reactions are detrimental for capacity loss, as the ratio *ρ* of typically <1 between capacity loss and coulombic consumption. The remaining electron transfer across the interface must be charge-neutralized by consuming charge inventory in the electrodes, as known as self-discharge process. On the other hand, in the full cell operation, a global charge inventory compensation is activated by the synergistic self-discharge processes between the positive electrode with electrochemical Li inventory and the negative electrode with electrochemical Li vacancies. A balanced coulombic consumption between the positive electrode and the negative electrode will play a positive role in improving the capacity retention of the battery. Accordingly, a mathematical approach was demonstrated using Gr||NMC532 model chemistry to quantitatively connect coulombic consumption and practical battery life. Further validation was conducted in multiple Si-based LIB chemistries to demonstrate two dimensionless and measurable parameters, the detrimental factor *ρ* and the balancing factor *i*_*p*_*/i*_*n*_, to be practically used for guiding the design of long-life LIBs batteries.

This study provides key insights into accurately implementing the measured coulombic loss in developing LIBs and beyond. It is critically argued that pursuing 99.98% CE is a utopian goal for high-performance batteries of 80% capacity retention for 1000 cycles. The realistic gap lies in the inevitable inventory burden from coulombic consumption in the parasitic reactions and its global compensation mechanism between the positive electrode and the negative electrode, no matter how hard we push the limit of suppressing the detrimental factors for better battery performance. It is demonstrated that incorporating physically meaningful criteria, namely *ρ* and *i*_*p*_*/i*_*n*_, can better guide the optimizations of LIBs chemistries. The balanced coulombic consumption (*i*_*p*_*/i*_*n*_) with global inventory compensation is a self-regulation mechanism to minimize continuous Li loss without the help of prelithiation and also inhibit voltage slippage problems without aggravated material degradation in the positive electrode at an ever-elevating voltage. Enhancement strategies like electrolyte additives should also pay attention to the working efficiency, which is the charge inventory burden to suppress the loss of active material, as described by the *ρ*. Moreover, our findings layout physical fundamentals for developing accelerated evaluation protocols. This physically reconstructed coulombic loss model, which relies on the law of charge conservation and the semi-empirical governing equations, will serve as one of the vital physical principles for the development of physics-informed neural networks (PINNs). In comparison to traditional data-driven machine learning, PINNs emphasize the constraints of physical principles to enhance the training process by significantly reducing lengthy data dimensions and expensive computation, leading to faster and more accurate performance evaluation and prognostics to accelerate battery innovation.

## Methods

### Materials and cell assembling

The electrode laminates of Gr (from Hitachi), NMC532 (from Toda Inc.), Si (from El-Cat), LFP (from Johnson Matthey), NMC811 (from Targray) materials were prepared at Argonne’s Cell Analysis, Modeling, and Prototyping (CAMP) Facility. For the coupled Gr |NMC532 full cells, Gr negative electrode contained 91.83 wt.% active material with 2 wt.% C45 conductive agent and 5 wt% Kureha 9300 binder and 0.17 wt% oxalic acid with active material loading of 6.4 mg cm^−2^, and NMC532 positive electrode contained 90% active material with 5 wt.% C45 and 5 wt.% Solvey-5130 polyvinylidene difluoride (PVdF) binder, with active material loading of 11.2 mg cm^−2^. For the coupled nSi||NMC811 full cells, Si negative electrode contained 80 wt.% active material with 10 wt.% C45 conductive agent and 10 wt% P84 polyimide binder with an active material loading of 0.9 mg cm^−2^, and NMC811 positive electrode contained 90% active material with 5 wt.% C45 and 5 wt% PVdF binder, with a material loading of 14.2 mg cm^−2^. The LFP electrode contains 90 wt.% LFP powder with 5 wt.% C45 conductive agent and 5 wt.% PVdF binder, and the NMC811^b^ electrode contains 96% NMC powder with 2 wt.% C45 and 2 wt.% PVdF binder^54^. The negative electrode capacity to positive electrode capacity (NP) ratio of 1.1 was utilized for all full cell assembly. All laminates were vacuum dried at 110 °C for 4 h to remove moisture prior to cell assembly. The stainless-steel CR2032 coin cells for fundamental investigations on coulombic loss and rapid HpLC tests contained 1.4-cm diameter positive electrode (an area of 1.539 cm^2^), 1.5-cm diameter negative electrode (1.767 cm^2^), or 1.6-cm diameter and 100-µm thick Li metal foil, with the Celgard 2325 separator. The 37-mAh large-format pouch cells for conceptual demonstration used 3.13 cm × 4.5 cm positive electrode (14.085 cm^2^) and 3.24 cm × 4.6 cm negative electrode (14.904 cm^2^). All electrode laminates are single-layer coating, and the overhang of negative electrode is about 0.5 mm in both coin cells and pouch cells. The external pressure of 26 psi was applied on pouch cells using the carbon-steel spring (part# 965fk353). The baseline liquid electrolyte in Gr||NMC532 cells was 40 μL of Gen2 electrolyte from Tomiyama Chemical Industry (1.2 M LiPF_6_ in EC/EMC, 3:7 by weight). The baseline liquid electrolytes in Si cells were 40 μL of Gen2 formula with 0%, 4%, and 10% FEC. The electrolyte amount in pouch cells is 1.5 µL mg^−1^ per electrode mass loading. Four localized high-concentration electrolytes (LHCEs) investigated for nSi||NMC811^a^ cells were (i) LiFSI-DMC-TTE (1:2.2:3 by mol), denoted as LHCE1, (ii) LiFSI-TMS-TTE (1:3:3 by mol), denoted as LHCE2, (iii) LiFSI-trimethyl phosphate (TMP) - TTE (1:1.4:3 by mol), denoted as LHCE3, and (iv) LiFSI - 1,2-dimethoxyethane (DME) - TTE (1:1.1:3 by mol), denoted as LHCE4. The assembled cells rested for 20 h at room temperature for sufficient wetting before formation cycles.

### Routine electrochemical characterization

Electrochemical cycling tests were carried out on a Landt battery testing system (CT3001A-5V1mA). The cycling protocol of full cells was performed at C/10 current rate at room temperature. The voltage windows for Li||Gr, Li||NMC532, and Gr||NMC532 half cells were 0.05-1.5 V, 3-4.3 V, and 2-4.25 V, respectively. The full cells were tested for 1000 cycles at 1 C after 2 formation cycles at C/10, followed by 5 cycles at C/10. The voltage windows for Li||Si, Li||NMC811, and Si||NMC811 half cells were 0.05–1.5 V, 3–4.2 V, and 2–4.15 V, respectively. The voltage windows for Li||LFP, and Si||LFP cells were 3–3.8 V, and 2–3.75 V, respectively. The cycling protocol with the napping procedure (denoted CC-CV-OCV) in Fig. 6d, e first followed 10 formation cycles at C/10 in 2.5–4.2 V and then followed a sequence of constant-current (CC) charging to 4.2 V at C/10, constant-voltage (CV) holding at 4.2 V for 1 h, and the open-circuit-voltage (OCV) napping or resting from 4.2 V for 1 h, and then constant-current discharge to 2.5 V at C/4 in every cycle till completing 100 cycles. The control cycling protocol followed the above procedure, except the OCV napping step before discharging. All reported cycling data in this study were collected from three repeating coin cells or two repeating large-format pouch cells at the environmental temperature in the lab. The current rates in all electrochemical tests follow the individual cell chemistry: Gr||NMC532 chemistry (1C_Gr||NMC532_ = 2.72 mA, 1C_Li||NMC532_ = 2.72 mA, 1C_Li||Gr_ = 3.68 mA), µSi||NMC811^b^ chemistry (1C_µSi||NMC811b_ = 6.56 mA, 1C_Li||NMC811b_ = 6.56 mA, 1C_Li||µSi_ = 8.07 mA), µSi||LFP chemistry (1C_µSi||LFP_ = 6.62 mA, 1C_Li||LFP_ = 6.62 mA, 1C_Li||µSi_ = 8.07 mA), nSi||NMC811^a^ chemistry (1C_nSi||NMC811a_ = 4.01 mA, 1C_Li||NMC811a_ = 4.01 mA, 1C_Li||nSi_ = 5.03 mA) and its pouch cell configuration (1C_nSi||NMC811a pouch cell_ = 37.24 mA).

### Open-circuit-voltage (OCV) aging

The calendar (OCV) aging tests of Gr||NMC532 began with 2 formation cycles at C/10, charging at C/10 to their highest SOC potentials in the defined voltage windows, followed by open-circuit voltage aging for an extended time (i.e., 1, 2, 3, and 6 months) at 30 °C, then discharging at C/10 to retrieve the remaining charge after aging, and finally using the cycling protocols for 3 cycles at C/10. Four repeating cells were assembled for each OCV testing condition. After OCV reference performance tests for 3 months, the positive and negative electrodes in full cells were harvested and remodeled in half cells using fresh Li metal counter electrode and fresh electrolyte for post diagnosis using their half-cell protocols. The harvested positive or negative electrodes in half cells were first discharged to quantify the loss of charge inventory in both electrodes (see Supplementary Fig. 2 for the voltage profiles of cells at different stages). The calendar (OCV) aging tests of Si-based cells began with 5 formation cycles at C/10 for stabilizing Si negative electrode, charging at C/10 to their highest SOC potentials in the defined voltage windows, following by open-circuit voltage aging for an extended time (i.e., 0.5 and 1 months), then discharging at C/10 to retrieve the remaining charge after aging, and finally using the cycling protocols for 5 cycles at C/10. All reported OCV-aging data in this study were collected from three repeating coin cells or two repeating large-format pouch cells in a temperature chamber at 30 ^o^C.

### High-precision leakage current measurement

Leakage current measurements were carried out on a high-precision leakage current (HpLC) system using Keithley 2401 source meter to measure coulombic consumption by parasitic reactions as a function of time. Keithley 2401 source meter has a voltage accuracy of 100 µV for the range of 20 V and, for current measurements, has 10 nA accuracy for 1 mA range and 100 pA accuracy for 10 µA range. The HpLC protocol began with 2 formation cycles at C/10 rate, presuming that a representative SEI was successfully formed on the electrode surface, and then iterated measuring steps of charging at C/10 to the targeted potentials and constant voltage holding for 300 h to measure the leakage current. All reported HpLC data in this study were collected from three repeating coin cells in a temperature chamber at 30 °C.

## Supplementary information

Supplementary Information

Peer review File

## Source data

Source data

## Data Availability

The data generated in this study are provided in the Supplementary Information and Source Data file. Source data are provided with this paper.
