# Supplementary material for: Deciphering coulombic loss in lithium-ion batteries and beyond
Source: Nat Commun. 2025 Jul 1;16:5785. doi: 10.1038/s41467-025-60833-y (PMC12216820; doi:10.1038/s41467-025-60833-y)
Supplement: Supplementary file 1 — Supplementary Information [file 41467_2025_60833_MOESM1_ESM.pdf]

Supplementary Information

Deciphering Coulombic Loss in Lithium-Ion Batteries and Beyond

Jiyu Cai,[1] Steve E. Trask,[1] Zhenzhen Yang,[1] Yingying Xie,[1] Wenquan Lu,[1] Hoai Nguyen,[1] Yuzi Liu,[2] Xiangbo Meng,[3] Gabriel M. Veith,[4] Hao Jia,[5] Wu Xu,[5] and Zonghai Chen[1,*]

[1] Chemcial Sciences and Engineering Division, Argonne National Laboratory, Lemont, IL 60439, USA

[2] Center for Nanoscale Materials, Argonne National Laboratory, Lemont, Illinois 60439, USA

[3] Department of Mechanical Engineering, University of Arkansas, AR 72701, USA

[4] Chemical Sciences Division, Oak Ridge National Laboratory, Oak Ridge, TN 37831, USA

[5] Energy and Environmental Directorate, Pacific Northwest National Laboratory, Richland, WA 99354, USA

* Correspondence: zonghai2025@yahoo.com

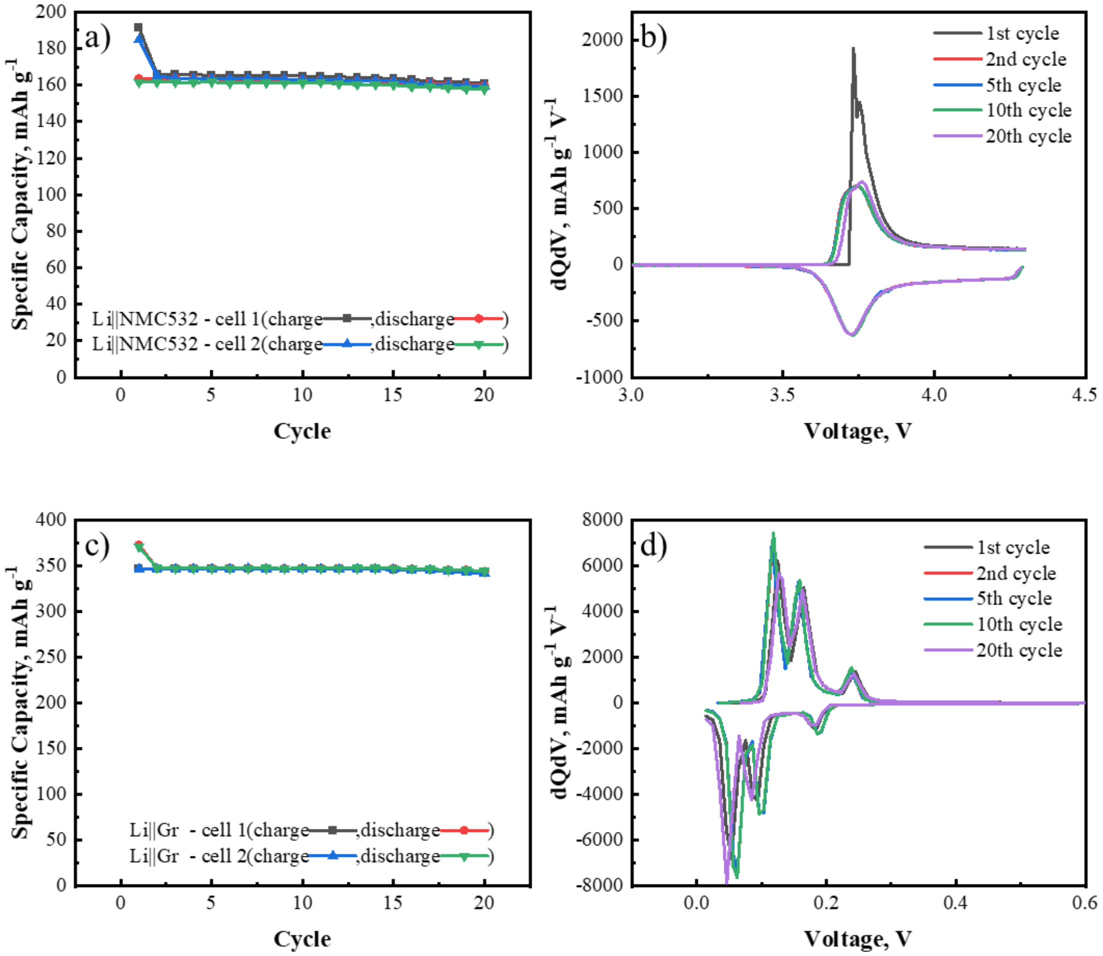

Supplementary Fig. 1. Electrochemical evaluation of NMC532 positive electrode and Gr negative electrode to be used in the model chemistry. Electrochemical cycling tests of (a) a Li||NMC532 half cell in voltage window of 3-4.3V at C/10 current rate and (c) Li||Gr half cell in 0.01-1.0 V at C/10. The dQdV plots of (b) Li||NMC532 and (d) Li||Gr at different cycles. The cycling tests were carried out at C/10 current rate ($1C_{Li||NMC532}$ = 2.72 mA, $1C_{Li||Gr}$ = 3.68 mA).

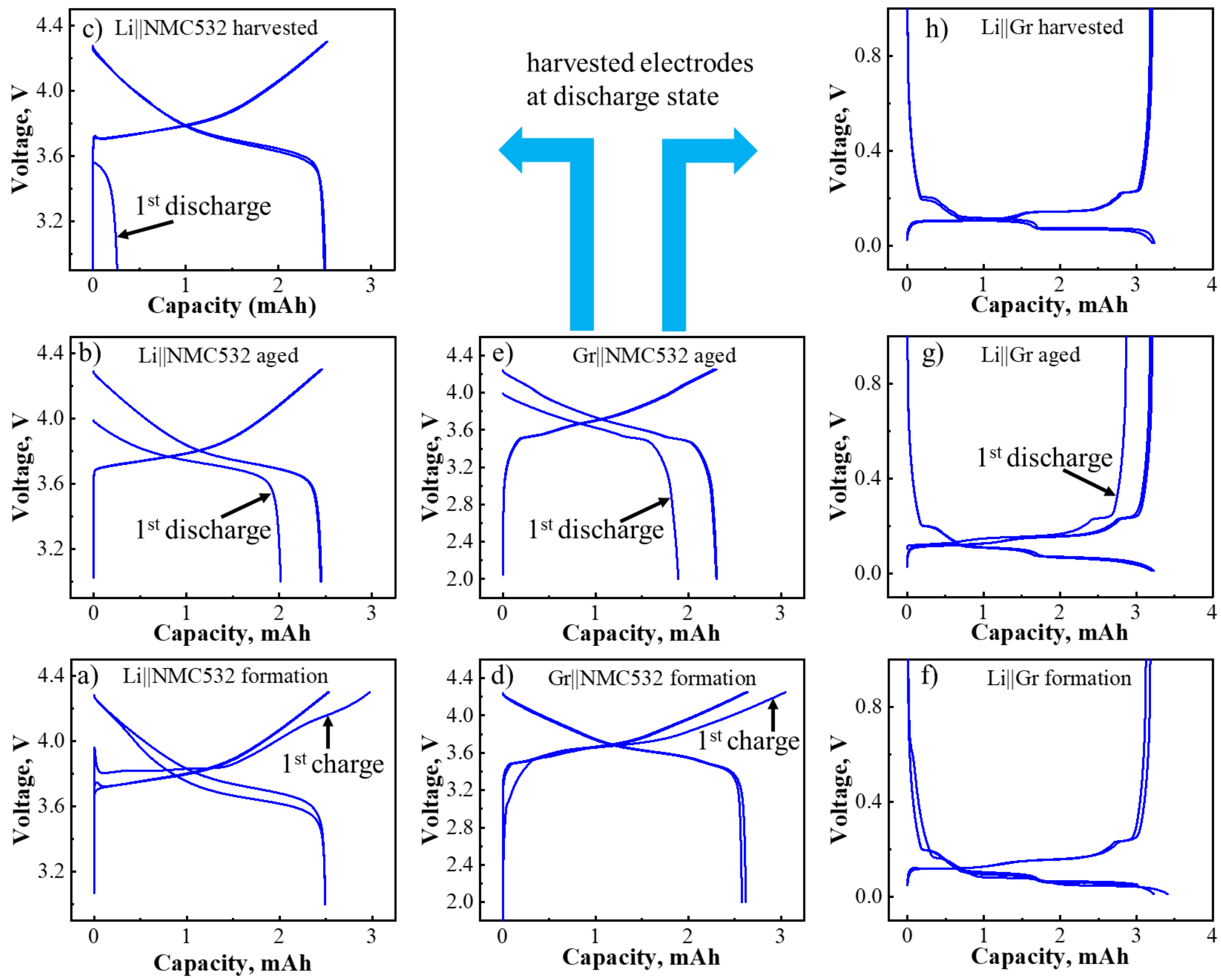

Supplementary Fig. 2. The OCV-RPT experiments of Gr||NMC532 model chemistry and the evaluations of aged behavior in full cells and individual electrodes. The voltage profiles of (a, b) Li||NMC532 half cell, (f, g) Li||Gr half cell, and (d, e) Gr||NMC532 full cell in the formation cycles and after 3-month OCV aging, respectively. The voltage profiles of (c) the harvested Li||NMC532 half cell and (h) the harvested Li||Gr half cell from the aged full cell. All electrochemical evaluations were carried out at C/10 current rate ($1C_{Gr||NMC532}$ = 2.72 mA, $1C_{Li||NMC532}$ = 2.72 mA, $1C_{Li||Gr}$ = 3.68 mA).

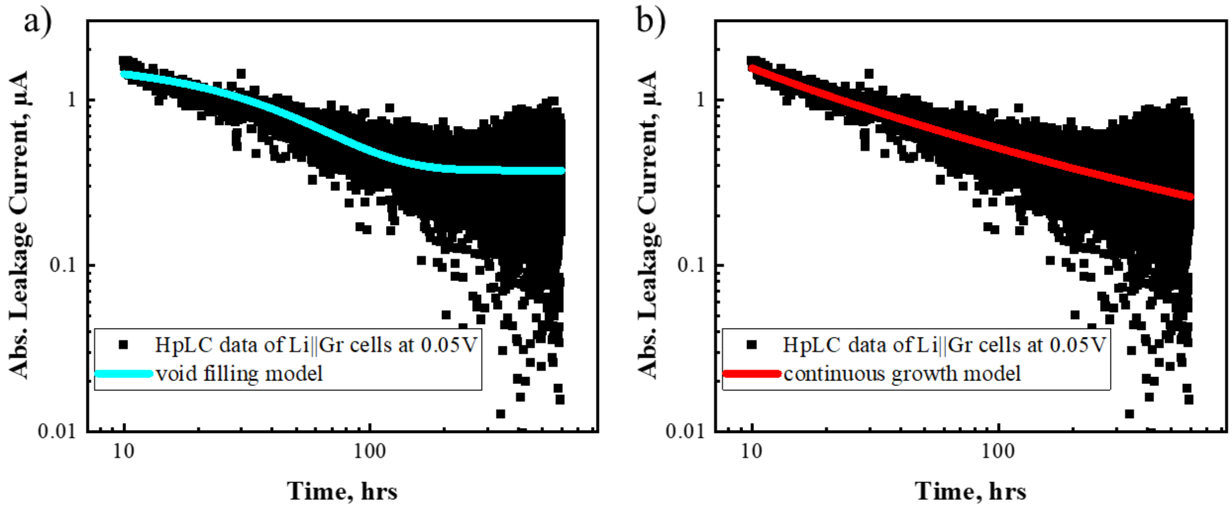

Supplementary Fig. 3. The descriptors for approximating the rate of electrolyte decomposition. Fitting for HpLC data of Li||Gr half cells at 0.05V using (a) void-filling model and (b) continuous-growth model.

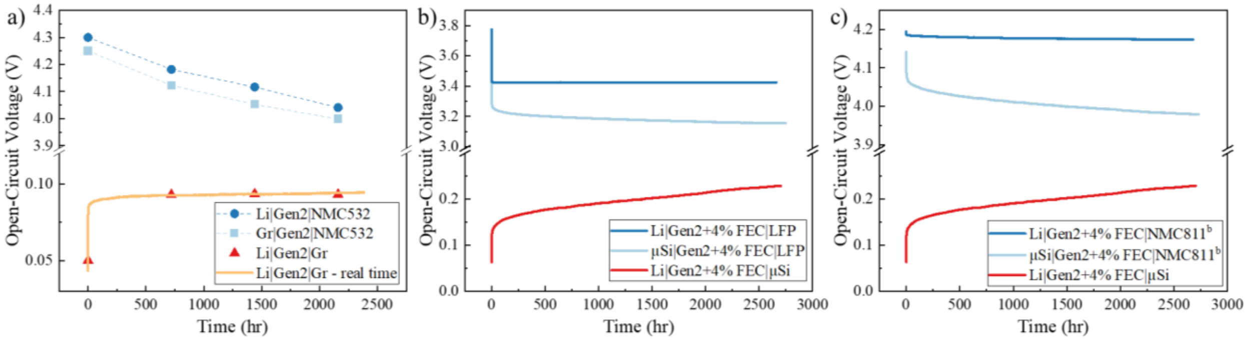

Supplementary Fig. 4. Voltage measurements of continuous self-discharge behavior over OCV aging time. The time evolution of open-circuit voltage in a) Gr||NMC532 full cells and their corresponding half cells, b) µSi||LFP full cells and their corresponding half cells, and c) µSi||NMC811[b] full cells and their corresponding half cells during calendar aging over 3 months. The discrete voltage values were measured for in a) Gr||NMC532 full cells and their corresponding half cells, which were disconnected from the battery cycler during OCV aging. The solid-line voltage curves were measured for the cells still connected on the battery cycler during OCV aging to record the real-time voltage evolutions. The internal impedance of Landt Instruments battery cycler (CT3001A-5V1mA) is higher than 1GΩ to measure accurate OCV values. The well overlap between the discrete data and the real-time curve in a) Li||Gr half cells indicates the reliable real-time voltage measurements.

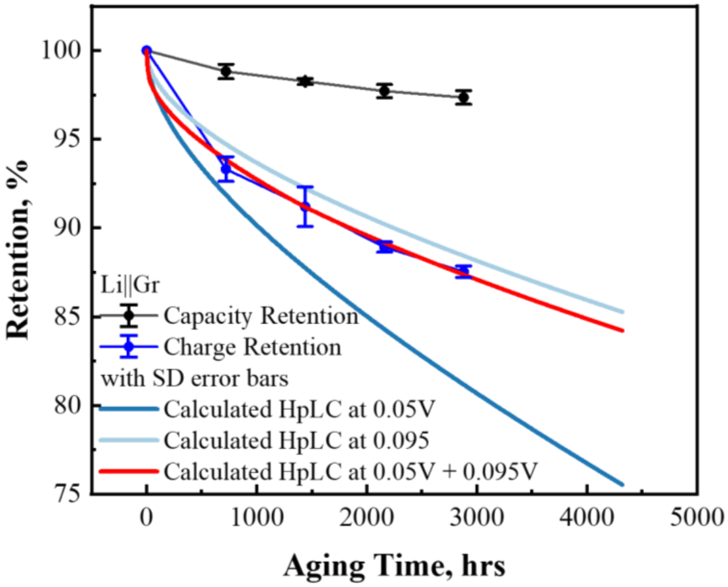

Supplementary Fig. 5. Approximations of time-evolving self-discharging behavior of Gr negative electrode from the calculation of rapid HpLC measurements. Calculated charge retention using HpLC data of Li||Gr half cells at 0.05 V or 0.095 V using continuous growth model and the combination of two sets of data, in the comparison with the measured OCV-RPT charge retention and capacity retention up to 4 months. The measured capacity and charge retentions show the mean value of three repeating cells in the center with SD error bars.

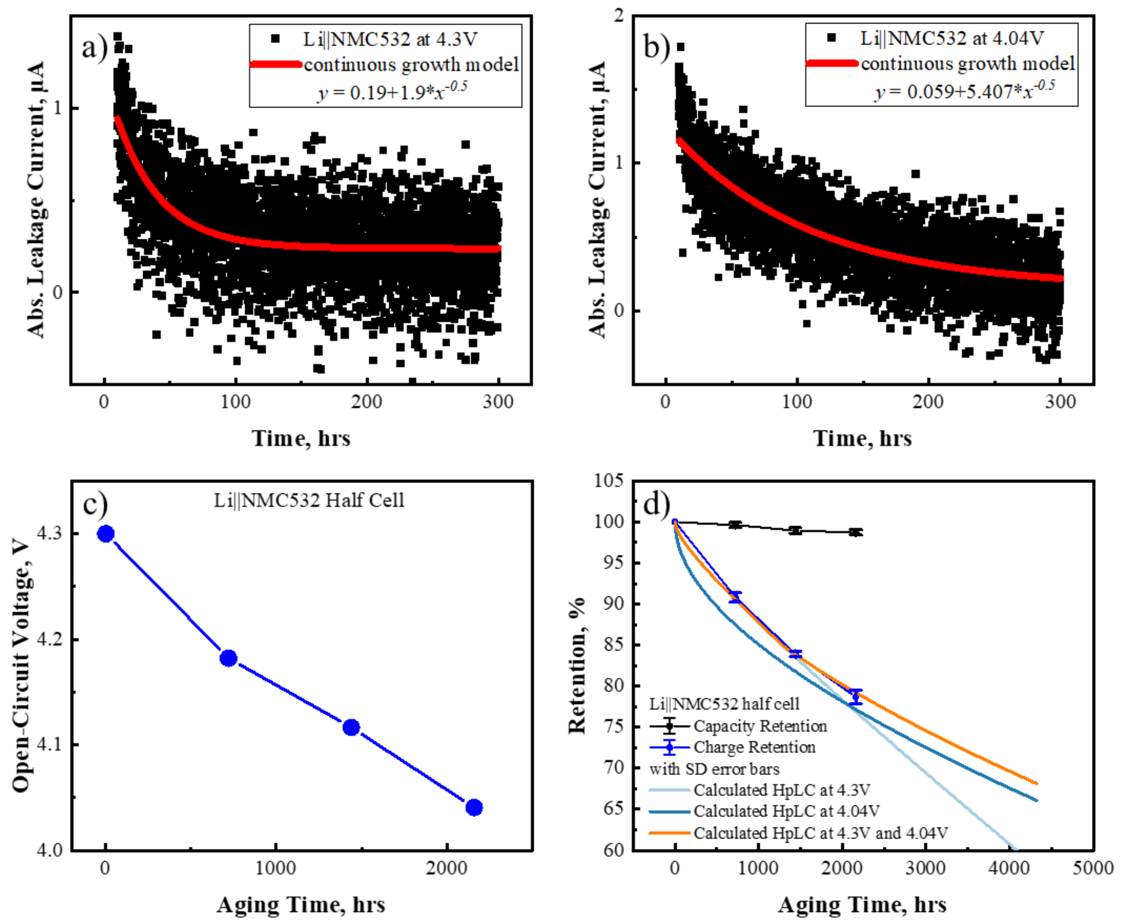

Supplementary Fig. 6. Approximations of time-evolving self-discharging behavior of NMC positive electrode from the calculation of rapid HpLC measurements. Fitting for HpLC data of Li||NMC532 half cells at (a) 4.3 V and (b) 4.04 V using continuous growth model. (c) The OCV evolution of Li||NMC532 half cells during calendar aging, initially from 4.3 V to 4.04 V after 3-month OCV aging. (d) Calculated charge retention using HpLC data of Li||NMC532 half cells at 4.3 V or 4.04 V using continuous growth model and the combination of two sets of data, in the comparison with the measured OCV-RPT charge retention and capacity retention up to 3 months. The measured capacity and charge retentions show the mean value of three repeating cells in the center with SD error bars.

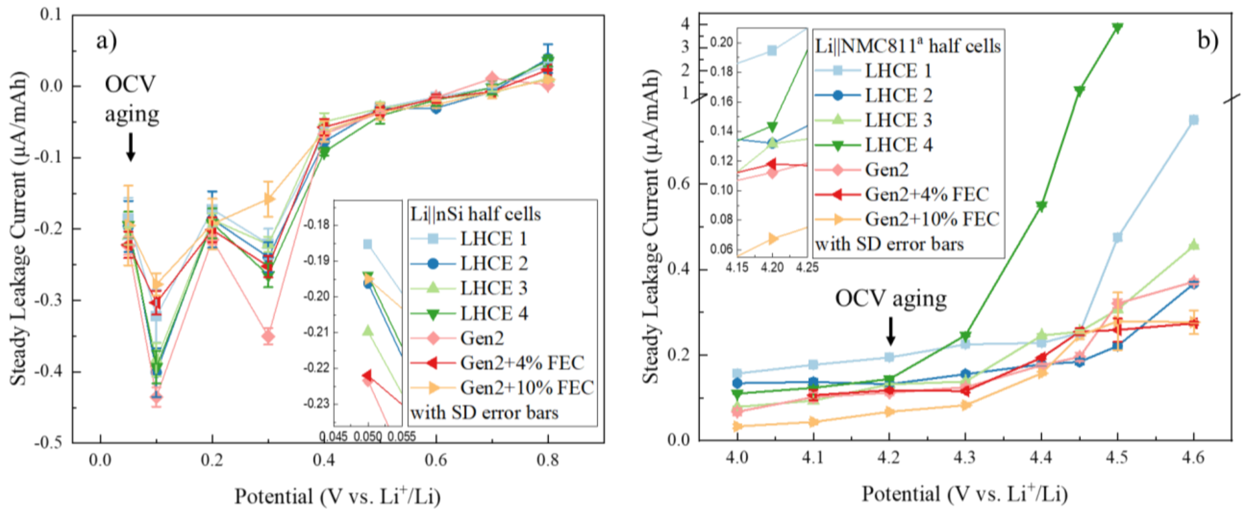

Supplementary Fig. 7. Electrolyte-dependent charge consumption rates on positive or negative electrodes for optimizing the global charge compensation mechanism. The rate of a) reductive parasitic reactions (i_n) at Si negative electrode and b) oxidative parasitic reactions (i_p) at NMC positive electrode as a function of their corresponding voltage range via HpLC measurments. The i_p/i_n ratio of each chemistry in the Figure 5a was calculated for their approximate voltage (i.e. 0.05V at Si negative electrode and 4.2V at NMC positive electrode) as OCV aging begins. The steady leakage current show the mean value of three repeating cells in the center with SD error bars.

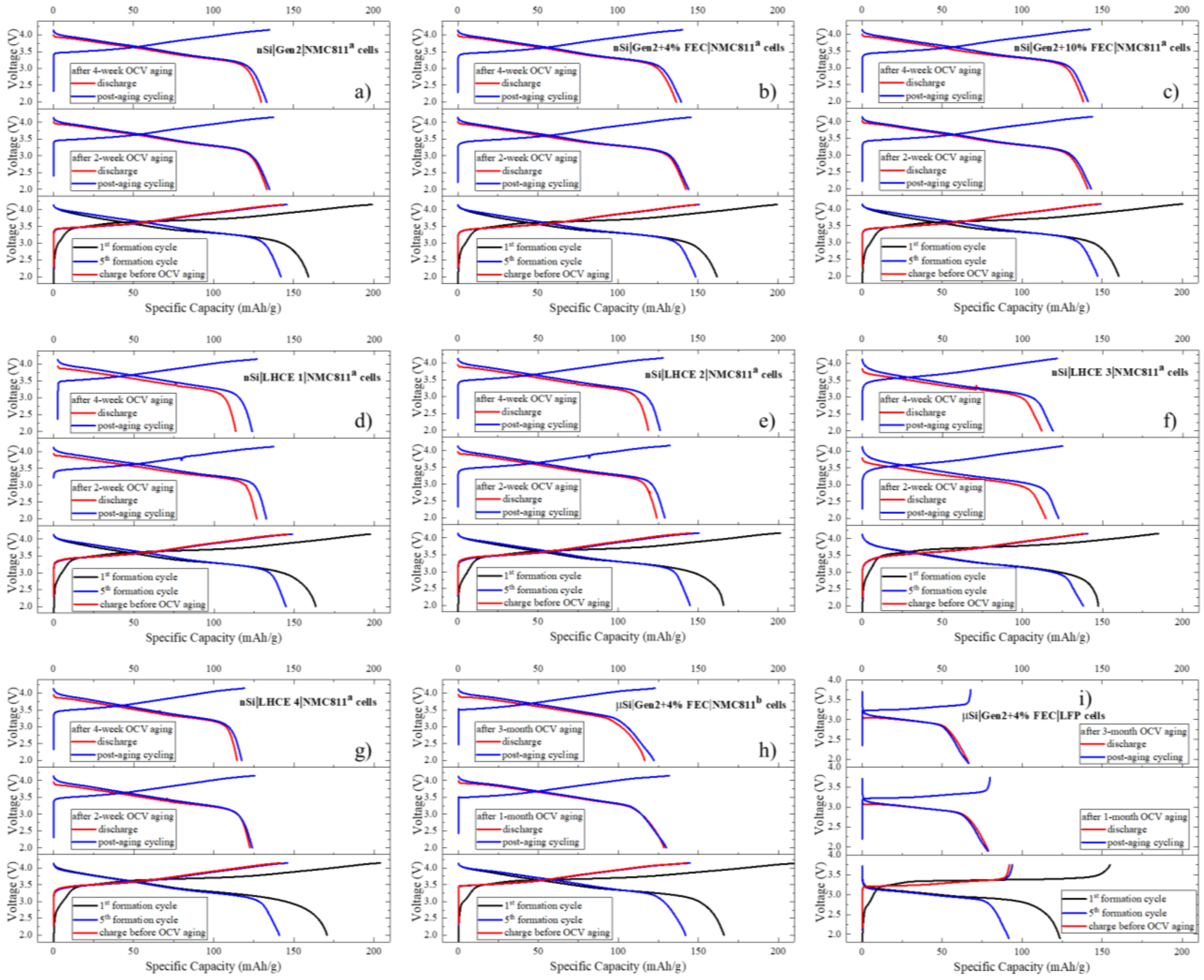

Supplementary Fig. 8. OCV-RPT experiments across multiple chemistries for demonstrating rapid screening. Voltage profiles of multiple Si-based lithium-ion battery chemistries during calendar aging tests, including a) nSi|Gen2|NMC811[a], b) nSi|Gen2+4%FEC|NMC811[a], c) nSi|Gen2+10%FEC|NMC811[a], d) nSi|LHCE1|NMC811[a], e) nSi|LHCE2|NMC811[a], f) nSi|LHCE3|NMC811[a], g) nSi|LHCE4|NMC811[a], h) µSi|Gen2+4%FEC|NMC811[b], and i) µSi|Gen2+4%FEC|LFP full cells. Each plot represents one of three repeating coin cells. The electrochemical tests in the formation cycles and post-aging cycles were all carried at C/10 current rate ($1C_{\mu Si||NMC811b}$ = 6.56 mA, $1C_{\mu Si||LFP}$ = 6.62 mA, $1C_{nSi||NMC811a}$ = 4.01 mA).

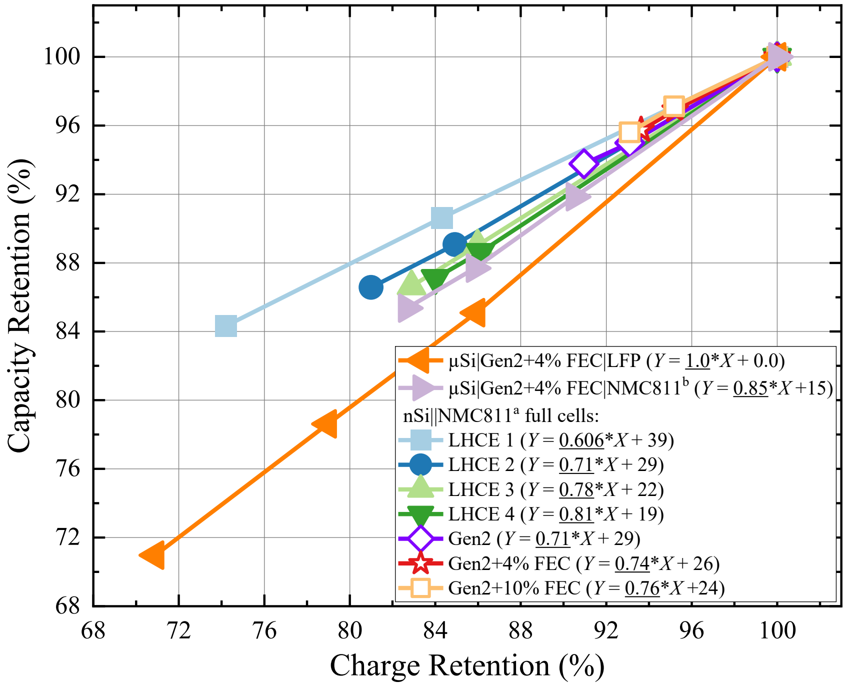

Supplementary Fig. 9. Determining the electrolyte-dependent detrimental ratio ρ from OCV-RPT experiments. Linear relationship between the measured capacity retention and the charge retention for multiple Si-based lithium-ion battery chemistries during calendar aging. Each data point represents the statistical data of three repeating coin cells.

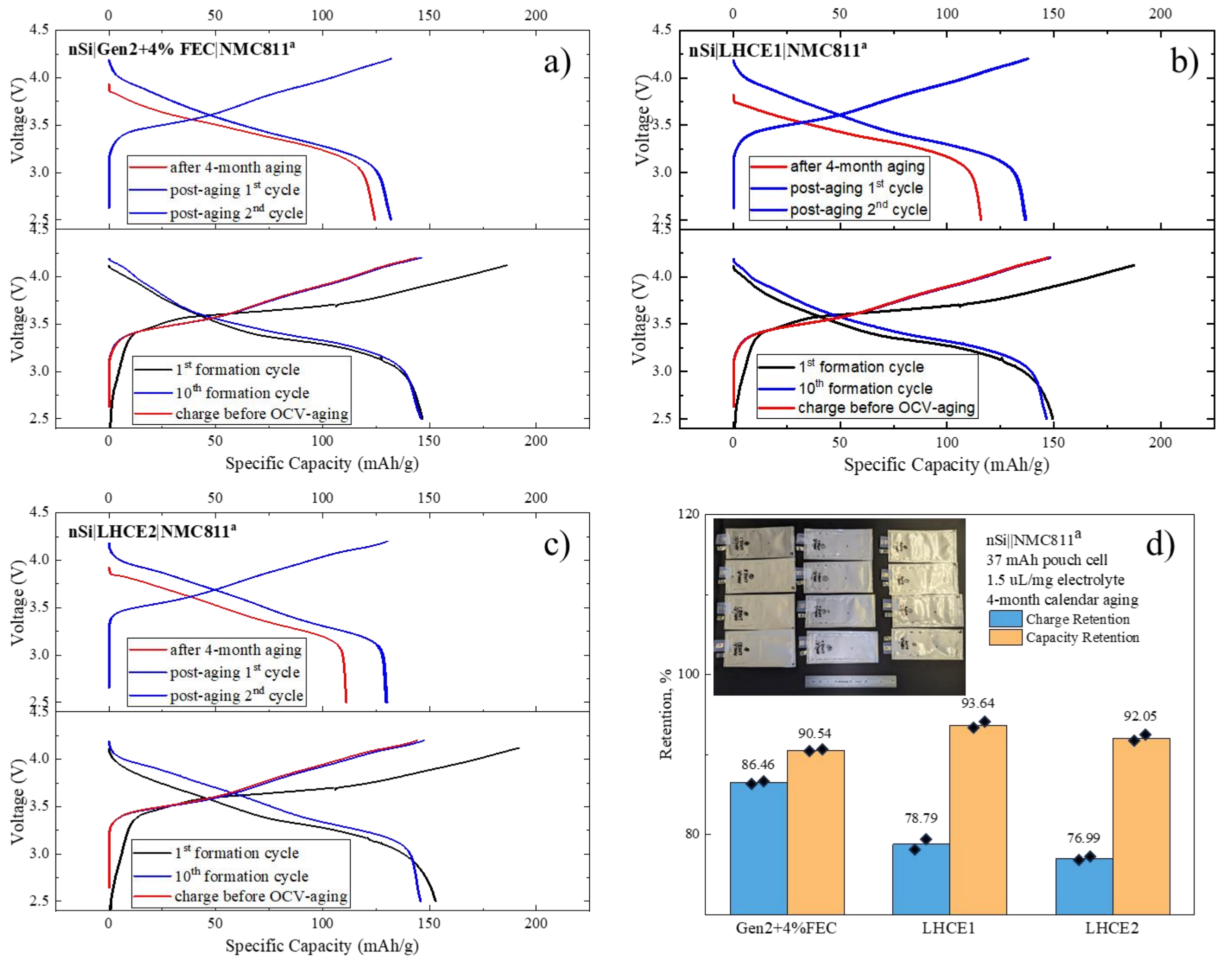

Supplementary Fig. 10. Conceptual demonstrations of physics-informed charge consumption model for rapid screening of optimal chemistry. (a-c) Voltage profiles of 37-mAh large-format nSi||NMC811[a] pouch cells using a) Gen2+4% FEC, b) LHCE1, and c) LHCE2 electrolytes, before and after 4-month calendar aging. (d) The comparison of charge retention and capacity retention during calendar aging using the mean values from two repeating pouch cells. The electrochemical tests were carried out at C/10 current rate ($1C_{nSi||NMC811a\ pouch\ cell}$ = 37.24 mA).

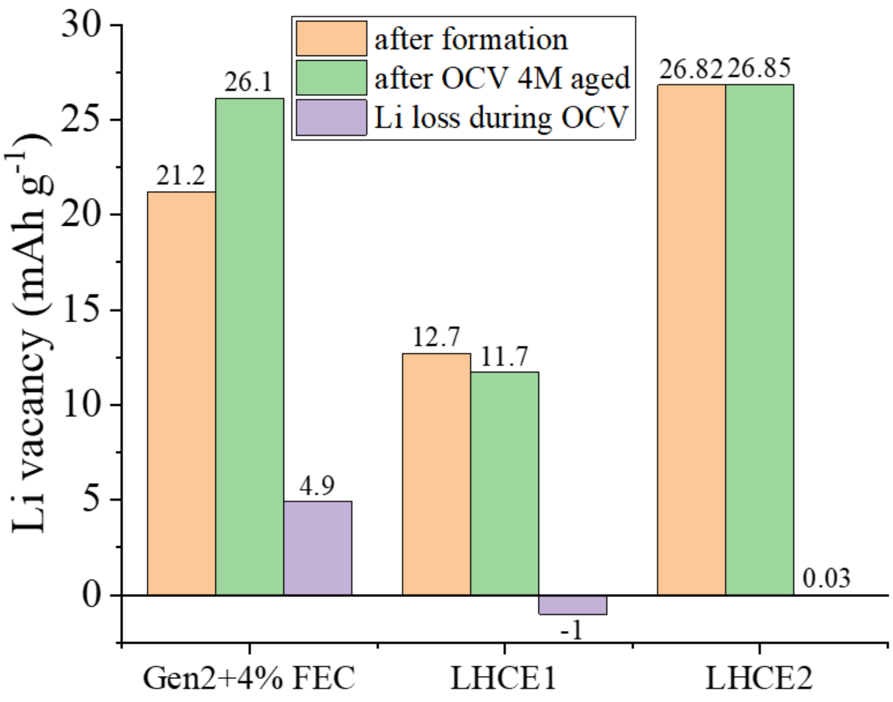

Supplementary Fig. 11. Experimental validation of the suppressed Li loss by global charge compensation mechanism. The electrolyte-determined Li vacancies in the 1st discharge process of harvested positive electrodes from full cells after formation cycles and 4-month OCV aging. The electrolyte of balanced charge consumptions (i_p/i_n) shows the negligible Li loss during OCV aging, due to the global charge inventory compensation.

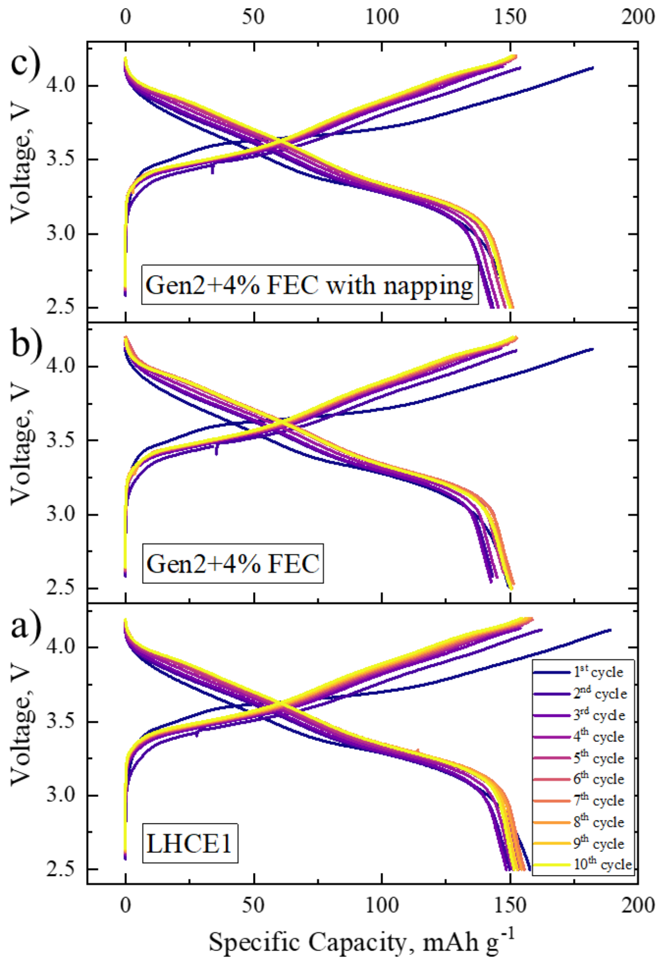

Supplementary Fig. 12. Electrochemical evaluations of the comparable performance between the different cell chemistry or in the same cell chemistry before different cycling protocols. The voltage profiles of nSi||NMC811[a] pouch cells using a) the i_p/i_n-balanced LHCE1 and b-c) the i_p/i_n-unbalanced Gen2+4% FEC electrolytes in the 10 formation cycles before cycling tests (corresponding to Figure 6d). A regular cycling protocol of CC-CV step was subsequently conducted on the cells denoted both a) LHCE1 and b) Gen2+4% FEC, while a modified cycling protocol of an extra OCV step after each CC-CV charging step was particularly performed on the cell denoted as b) Gen2+4% FEC cells with napping. These demonstrate the significant contribution of global charge compensation from the i_p/i_n-optimized chemistry or the napping procedure in the same chemistry to enhance cell performance.

Supplementary Table 1. Summary of cycling performance of Gr||NMC532 cells in various chemistries.

Manufactor	Electrolyte	Modification	Voltage V	Temperature ºC	Cycling Performance	Capacity Fading %/cycle	Ref
CAMP	1.2M LiPF6 in EC/EMC (3:7)	none	4.2	R.T.	73% - 1000 cycles	0.027	this study
lab	1.2 M LiPF6 in EC:DEC (3:7)	none	4.2	30	80% - 1000 cycles	0.020	1
lab	1.2M LiPF6 in EC/EMC (3:7)	none	4.2	-	70.5% - 500 cycles	0.119	2
lab	1.2M LiPF6 in EC/EMC (3:7)	none	4.2	30	74% - 1000 cycles	0.026	3
LiFun	1M LiPF6 in EC/EMC (3:7)	none	4.4	40	90% - 300 cycles	0.033	4
LiFun	1.5M of LiPF6 in EC/EMC/DMC (25:5:70)	none	4.3	40	80% - 500 cycles	0.040	5
LiFun	1.5M of LiPF6 in EC/EMC/DMC (25:5:70)	none	4.3	40	80% - 300 cycles	0.067	6
LiFun	1.2M LiPF6 in EC/EMC (3:7)	2%FEC additive	4.2	40	96% - 400 cycles	0.010	7
LiFun	1.5M LiPF6 in EC/DMC (3:7)	2% VC additive	3.8	40	95% - 1000 cycles	0.005	8
LiFun	1.1M LiPF6 in EC/EMC (3:7)	2%VC + 1%DTD additives	4.3	40	95% - 900 cycles	0.006	9
LiFun	1.5M LiPF6 in EC/EMC (3:7)	2%VC + 1%DTD additives	4.2	40	95% - 1000 cycles	0.005	10
LiFun	1.2M LiPF6 in EC/EMC (3:7)	Al2O3 coating, VC211 additives	4.2	20	90% - 1000 cycles	0.010	11

Supplementary Table 2. Summary of the measurable and transferable performance indicators, i_c or i_a for charge inventory compensation mechanism and the ρ for the charge-to-capacity correlation factor, in the 9 investigated LIB chemistries to show the validity and broadness of our hypothetical concepts.

Cell Chemistries	Positive/ Working Electrode	Negative/ Counter Electrode	Electrolyte	i μA/mAh	ρ
	LFP	μSi		-	1.00
1	LFP	Li	Gen2+4%FEC	0.017	1.02
	μSi	Li		0.150	0.89
	NMC811[b]	μSi		-	0.85
2	NMC811[b]	Li	Gen2+4%FEC	0.050	0.74
	μSi	Li		0.150	0.89
	NMC811[b]	nSi		-	0.75
3	NMC811[b]	Li	Gen2+4%FEC	0.118	0.87
	nSi	Li		0.310	0.74
	NMC811[a]	nSi		-	0.71
4	NMC811[a]	Li	Gen2	0.112	0.39
	nSi	Li		0.213	0.43
	NMC811[a]	nSi		-	0.76
5	NMC811[a]	Li	Gen2+10%FEC	0.067	0.91
	nSi	Li		0.195	1.26
	NMC811[a]	nSi		-	0.61
6	NMC811[a]	Li	LHCE1	0.194	0.12
	nSi	Li		0.190	0.24
	NMC811[a]	nSi		-	0.71
7	NMC811[a]	Li	LHCE2	0.132	0.18
	nSi	Li		0.200	0.57
	NMC811[a]	nSi		-	0.78
8	NMC811[a]	Li	LHCE3	0.132	0.12
	nSi	Li		0.210	1.05
	NMC811[a]	nSi		-	0.81
9	NMC811[a]	Li	LHCE4	0.144	0.22
	nSi	Li		0.190	0.86

* nSi denotes for nano-size Si, and μSi is micro-size Si
* LFP denoted for $LiFePO_4$
* Gen2 has the formula of 1.2 M $LiPF_6$ in EC/EMC (3:7 by weight)
* NMC811[a] and NMC811[a] are used for coupling nSi and μSi negative electrodes with different mass loadings, respectively. Their N/P ratios are 1.1.

Supplementary Note 1

Analytical solution for the charge balance model

It has been widely accepted that continuous parasitic reactions undergoing at the electrode/electrolyte interface are responsible for the loss of charge inventory and the loss of active electrode materials, both of which are major contributors to the performance loss of lithium-ion batteries (LIBs) during storage and cycling. Therefore, the loss of charge inventory, and/or the loss of active materials, have been widely and vaguely used to interpret the fading mechanism of LIBs. However, the analytical relationship among these factors is yet to be established.

Supplementary Fig. 13 shows the relationship between the charge retention and capacity retention of various cells (Li||NMC532, Li||Gr, and Gr||NMC532, with NMC532 standing for $LiNi_{0.5}Mn_{0.3}Co_{0.2}O_2$ and Gr for graphite) during the open circuit voltage (OCV) aging. The charge retention is a direct measurement of the loss of charge inventory during the OCV aging (charge retention + charge loss =100%), while the capacity retention is a direct measurement of the retention of reversible capacity after OCV aging. It is clearly shown in Supplementary Fig. 13 that the charge loss during aging has a linear contribution to the loss of reversible capacity regardless of the cell chemistry. However, the significant contribution of the charge loss to the capacity loss, the slope of the capacity retention to the charge retention, is highly dependent on the cell chemistry.

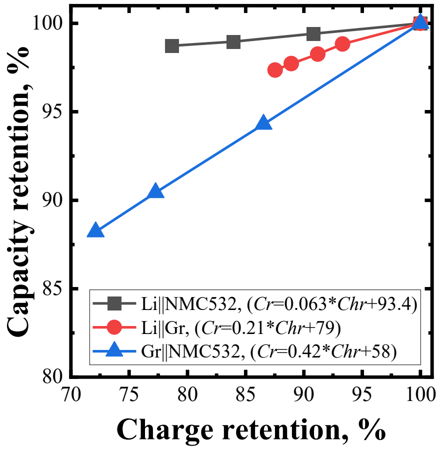

Supplementary Fig. 13. Relationship between charge retention and capacity retention for different cell chemistries.

Using Li||Gr as an example, the ratio of the capacity retention to the charge retention is measured to be about 0.21. It physically means that not all the loss of charge inventory equally and fully contributes to the capacity loss. Given the unlimited OCV aging, the Li||Gr cells can self-discharge to 0% state of the charge (SOC) and the parasitic reactions will be kinetically self-terminated, the Li||Gr can still be charged again and deliver 79% of its initial reversible capacity, leaving only 21% of its charge loss contributes to irreversible capacity loss. As schematically shown in Figure 3a, the loss of charge inventory can be physically measured using either OCV aging or high precision leakage current measurement (HpLC), or high precision coulombic efficiency (CE) measurement. However, its electrochemical impact must follow charge neutrality mechanisms for

several electron transfer reactions. On the Gr negative electrode, the parasitic reactions are fundamentally the reduction reactions of electrolyte components at the surface of negative electrode, resulting in transfer of electrons from the working electrode (lithiated graphite, Li_xC) to the electrolyte. To maintain the local charge neutrality, the electron transfer reaction needs to be compensated by the equivalent amount of alternative charges. One simple charge compensation mechanism is the loss of Li ions from the charged negative electrode (Li_xC), leaving an equivalent amount of active sites in the negative electrode that can reversibly accept lithium-ions in the following cycling. The portion of capacity is still considered reversible in half cells, but its reversibility is conditional on the availability of Li-ion reservoir in the counter electrode. The slope between the capacity retention to the charge retention (0.21) physically means that about 21% of the charge loss contributes to the physically loss of accessible active material, leading to a permanent loss of reversible capacity regardless of Li-ion inventory of the counter electrode. Based on the same principle for charge compensation, the electron transfer reactions at the cathode/electrolyte interface (CEI) also accompanies irreversible loss of accessible active materials and conditionally reversible charge loss. Supplementary Fig. 14 schematically shows the contribution of charge loss of the negative electrode and the positive electrode, individually, to the overall capacity retention of full cells. During the charging process, a certain amount of Li inventory will be removed from the positive electrode and inserted into the negative electrode. If there is no aging process by assuming both charge retention and capacity retention to be 100% for both the positive electrode and the negative electrode, the full cell can deliver 100% of its reversible capacity without any loss of accessible capacity (see Supplementary Fig. 14). In reality, parasitic reactions will independently consume Li inventory in the negative electrode and refill the vacancy in the positive electrode (see Figure 3a). If all charge loss is irreversible to capacity loss, like the slope ρ equal to 1, the capacity retention of the full cell (Cr_f) will be the smaller value between the charge retention of the negative electrode (Chr_n) and the charge retention of the positive electrode (Chr_p) as shown in the following equation.

$$Cr_f = min\ (Chr_n, Chr_p) \tag{1}$$

However, one can quickly find out that Equation 1 severely overestimates the impact of the loss of charge inventory at both the positive and negative electrodes. The root of this discrepancy is that not all charge loss of individual half cells, $1 - Chr$, will equally contribute to the capacity loss, $1 - Cr$ or $\rho * (1 - Chr)$, as discussed above for the practical slope ρ of <1. Except for the portion causing irreversible capacity loss, the remaining charge consumption in parasitic reactions is compensated by the loss of charge inventory in individual electrodes, $(1 - Chr) - \rho * (1 - Chr)$ or $(1 - \rho) * (1 - Chr)$. Herein, the self-discharge process will generate extra Li vacancy at the negative electrode, while the positive electrode during self-discharging will refill with extra Li inventory. The gained Li inventory from the self-discharging process in the positive electrode can be used to compensate the gained Li vacancy in the negative electrode for a global charge inventory compensation mechanism. One should note that the compensation amount is dependent on the minimum accessible charge inventory of either the Li supply in the positive electrode or the Li vacancy in the negative electrode. It is suggested that a balanced charge inventory between the positive electrode and the negative electrode will provide the most global inventory compensation

to improve the battery performance. Therefore, a mathematical approach can be established for the capacity retention of the full cells, including charge consumption by the parasitic reactions and the global charge inventory compensation between both electrodes.

$$Cr_f = min(Chr_n, Chr_p) + min\,((1-\rho_p) * (1-Chr_p), (1-\rho_n) * (1-Chr_n)) \qquad (2)$$

In above Equation 2, ρ_n is the ratio between the capacity retention and the charge retention of the negative electrode and ρ_p is the ratio between the capacity retention and the charge retention of the positive electrode (see Supplementary Fig. 13).

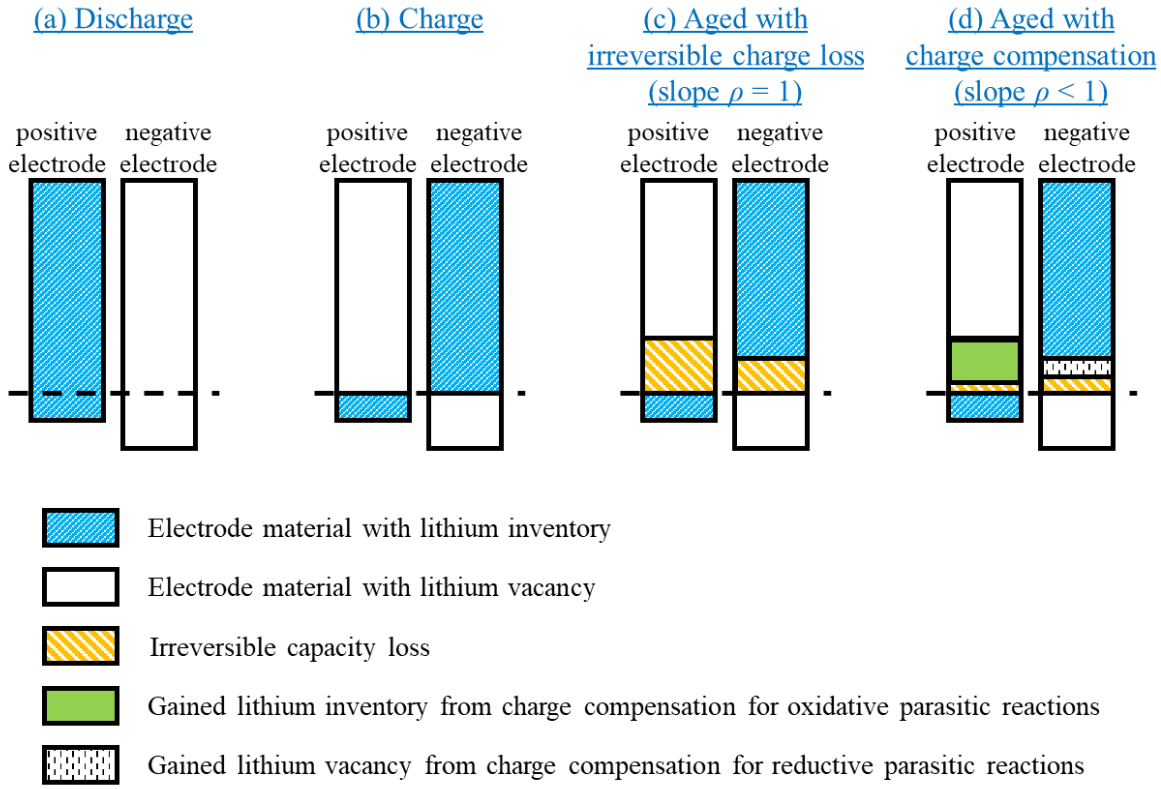

Supplementary Fig. 14. Schematics showing the important contribution of charge compensation to the capacity retention of full cells.

Supplementary references:

1 An, S. J., Li, J. L., Du, Z. J., Daniel, C. & Wood, D. L. Fast formation cycling for lithium ion batteries. *J Power Sources* **342**, 846-852, doi:10.1016/j.jpowsour.2017.01.011 (2017).

2 Nur, A. *et al.* Single-crystal LiMNC532 synthesized from mixed hydroxide precipitate (MHP) via co-precipitation-sintering route for enhanced lithium-ion battery lifespan. *J Phys Chem Solids* **200**, doi:ARTN 11261410.1016/j.jpcs.2025.112614 (2025).

3 Wood, M. *et al.* Impact of secondary particle size and two-layer architectures on the high-rate performance of thick electrodes in lithium-ion battery pouch cells. *J Power Sources* **515**, doi:ARTN 23042910.1016/j.jpowsour.2021.230429 (2021).

4 Li, J. *et al.* Comparison of Single Crystal and Polycrystalline LiNiMnCoO Positive Electrode Materials for High Voltage Li-Ion Cells. *Journal of the Electrochemical Society* **164**, A1534-A1544, doi:10.1149/2.0991707jes (2017).

5 Azam, S. *et al.* Impact of Electrolyte Additives on the Lifetime of High Voltage NMC Lithium-Ion Pouch Cells. *Journal of the Electrochemical Society* **171**, doi:ARTN 11051010.1149/1945-7111/ad8d0c (2024).

6 Song, W. T. *et al.* Lithium Difluoro(dioxalato) Phosphate as an Electrolyte Additive for NMC811/Graphite Li-ion Pouch Cells. *Journal of the Electrochemical Society* **169**, doi:ARTN 11051310.1149/1945-7111/ac96aa (2022).

7 Li, J. *et al.* Methyl Acetate as a Co-Solvent in NMC532/Graphite Cells. *Journal of the Electrochemical Society* **165**, A1027-A1037, doi:10.1149/2.0861805jes (2018).

8 Aiken, C. P. *et al.* Li[NiMnCo]O as a Superior Alternative to LiFePO for Long-Lived Low Voltage Li-Ion Cells. *Journal of the Electrochemical Society* **169**, doi:ARTN 05051210.1149/1945-7111/ac67b5 (2022).

9 Thompson, L. M. *et al.* Study of Electrolyte and Electrode Composition Changes vs Time in Aged Li-Ion Cells. *Journal of the Electrochemical Society* **168**, doi:ARTN 02053210.1149/1945-7111/abe1da (2021).

10 Li, J., Li, H. Y., Stone, W., Glazier, S. & Dahn, J. R. Development of Electrolytes for Single Crystal NMC532/Artificial Graphite Cells with Long Lifetime. *Journal of the Electrochemical Society* **165**, A626-A635, doi:10.1149/2.0971803jes (2018).

11 Li, J. *et al.* Effect of Choices of Positive Electrode Material, Electrolyte, Upper Cut-Off Voltage and Testing Temperature on the Life Time of Lithium-Ion Cells. *Journal of the Electrochemical Society* **165**, A3195-A3204, doi:10.1149/2.0931813jes (2018).
