# Supplementary material for: Deciphering coulombic loss in lithium-ion batteries and beyond
Source: Nat Commun. 2025 Jul 1;16:5785. doi: 10.1038/s41467-025-60833-y (PMC12216820; doi:10.1038/s41467-025-60833-y)
Supplement: Supplementary file 2 — Peer review File [file 41467_2025_60833_MOESM2_ESM.pdf]

nature portfolio

Peer Review File

Deciphering Coulombic Loss in Lithium-Ion Batteries and Beyond

Corresponding Author: Dr Zonghai Chen

This file contains all reviewer reports in order by version, followed by all author rebuttals in order by version.

Version 0:

Reviewer comments:

Reviewer #1

(Remarks to the Author)
This paper describes a model that takes into account various reactions in lithium-ion batteries, and predicts the battery life by analyzing the rate of side reactions determined from the self-discharge current during constant potential charging. The reviewer agrees with the authors' claim that the battery life does not correspond to the Coulombic efficiency but is related to the current efficiency of the positive and negative electrodes, and convincing evidence is presented in the paper. However, there are many ambiguities regarding the side reactions inside the battery, and more detailed explanations are necessary. Some unclear points are noted, and see my comments below.

1. Two parameters, ρ and ic/ia, are presented, but no correlation with capacity retention is shown. Is there any relationship between ic/ia in Fig. 5a and capacity retention in c?

2. The diagram in Fig. 3 seems suspicious and should be drawn more precisely. γe is thought to be an electron passing through an external circuit. If it is an electron in this equation, X -> X+ + e- as shown in the diagram, then γe must be the same as γX. Also, the equation for back insertion should involve the electrons as follows; MO2 + Li+ + e- -> LiMO2, because MO2 + Li+ -> LiMO2 in the diagram does not satisfy the electrical neutrality.

3. Please explain in detail the relationship between I(t) in Eq.10-14 and γe. In particular, since γe=0 in the aging state, γX is equal to γLi, because back insertion should be paired with a side reaction to form a local cell. Moreover, the reviewer thinks that the self-discharge current measured in the Li half-cell corresponds to γX at the positive and negative electrodes.

4. The authors' claim that the battery life does not correspond to the Coulomb efficiency but is related to the current efficiency of the positive and negative electrodes is also presented in the previous study below, which should be cited.

K. Nakura et al., J. Electrochem. Soc. 158 (2011) A1243–A1250.
J. Xu et al., J. Electrochem. Soc. 162 (2015) A2026–A2035.
R.D. Deshpande et al., J. Electrochem. Soc. 162 (2015) A330–A338.
K. Ariyoshi at al., Electrochemistry 87 (2019) 188–192.

5. In this paper, the authors do not discuss crosstalk reactions, which plays an important role on the side reactions inside the batteries. The reviewer is somewhat skeptical as to why this study was able to predict capacity retention without taking crosstalk responses into account. Please provide some comments on this.

Reviewer #2

(Remarks to the Author)
The paper under revision „Deciphering Coulombic Loss in Lithium-Ion Batteries and Beyond" provides a study on the physics behind coulombic loss, to ultimately provide a new method to predict lifetime.

The topic of lifetime testing and prediction is very relevant as testing takes a considerable amount of time and lifetime is a major contributor to the CO2 footprint as well as the total cost of ownership. In this study, a series of cycle and calendar tests were conducted as a reference for a method that uses voltage hold measurements to calculate characteristics that allow

cycle and calendar life to be modeled in 0.5 months for at least 6 months of conventional testing.
The results indicate considerable potential in reducing testing time, while still maintaining accurate predictions.
Below are several major and minor points that I think may improve the quality of the paper. Because of these points I would recommend major revisions before publication.

Major issues:
1. Implications from the anode overhang
a. When cells are stored at high SOC the geometrical anode overhang is filled which can only partly be extracted again. In lab cells the anode overhang can sometimes have significant contribution to capacity loss and or charge loss
i. Please provide information about the cell design anode and cathode area. Double side coating or only single side coating
ii. Please discuss the impact of anode overhang on the capacity loss and charge loss
2. Capacity retention and charge retention are very low for the investigated systems
a. i.e. figure 2 b) a capacity retention of < 90% after 3 months for the full cell at 4.2 V is found please compare this to reported data from commercial cells and comment on how the obtained performance of the investigated cells might influence the outcome with regard to state-of-the-art cells.
b. i.e. figure 1 b) same here for the capacity retention after cycling 1000 cycles led to 73% SOH
c. figure 1 b) please show the results from multiple cells we found strong deviations (100%) for this in our data from LiFun Cells
d. how would you explain such deviations?
e. please compare your found self-discharge of around 20% (figure 2 c ~0.5 mAh/2.5 mAh) to other publications on calendar aging
i. please provide OCV vs time data for the OCV tests.
f. please highlight the temperature under which each experiment was conducted
3. Can the authors please discuss on how the li metal will influence the half cell results, I was thinking on crosstalk especially for the impact of different electrolytes.
4. Statistics and error discussion
a. Please provide standard deviation and sample size for each reported data set.
b. Please provide voltage and current accuracy of the Keithley 2401 source meter
5. Please provide information on the napping procedure in the method section:
a. for how long was the rest phase
b. at which SOC
c. for how often?
6. The paper suggests a correlation of the charge balance parameter (ic/ia) and the cell performance indicator (ρf), please provide for all tested cell chemistries in Figure 5 a) the OCV-RPT data as well as cycling data. Give a quantitative analysis on which ic/ica range is beneficial and in which manner this is needed for further validation of the proposed method.
7. In my opinion, the standard aging test is more likely to be done by evaluating the discharge capacity/energy over the cycle as shown in Figure 5 d). Please discuss the advantages of your methods including the accuracy when considering measurement errors and compare it to the direct measurement as performed in Figure 5 d) when the same period of testing time is used in both methods. To my understanding, in the standard cycling test I will get direct quantitative results, while the new method is intriguing, it only results in parameters that correlate qualitatively with performance.
Minor issues:
8. Writing style. The clarity of the document could be improved:
a. Limiting sentences to maximum of 3 lines (i.e. page 3 “This investigation uncovered two dimensionless and measurable coulombic descriptors to quantitatively connect with battery capacity in physically meaningful manners: one being , which is defined as the “detrimental” ratio that partial by products of parasitic reactions are responsible for irreversible capacity fading of electrode materials, and the other, ic/ia, defined as the “balanced” ratio that coulombic consumptions between cathode and anode should be balanced to globally compensate the usable inventory in full cells.”
b. Avoid judgmental wording like “Large discrepancy” – “heavy overestimation”
c. Remove filling phrases
d. When results are presented it is helpful to give the found results in text and elaborate how the conclusion was drawn. (i.e. “The NMC532||Gr full cells after OCV-RPT tests for 3 months were dissembled to harvest NMC532 and Gr electrodes and prepare them in half cells using fresh Li metal anode and electrolyte. It is found that the electrochemical performance of harvested NMC532||Li and Gr||Li cells is identical to those aged in half-cell configuration (Figures 2c-e, Figure S4).” Here only a conclusion is given and the reader has to look at the data, assess by him/herself how that conclusion was drawn.)
9. The authors state several times that the community would rely on coulomb efficiency when developing new chemistries. I do not agree here. The main testing carried out is cycle life testing focusing on discharge capacities. This is not to state that the found quantification and modeling of the charge loss is less relevant, however, I find the motivation a little exaggerated.

Reviewer #3

(Remarks to the Author)
Quantitative relationship between the charge consumption at the electrode/electrolyte interface and the battery performance is crucial for understanding the performance degradation mechanism and providing guidance for rational design of high performance batteries. The authors identified a method to compare how OCV aging affects capacity loss and charge loss differently with high-precision static leakage current measurements of the anode and cathode (ic/ia) and used it to predict the capacity loss very accurately. Focusing on different charge consumption rate at the cathode and the anode, this manuscript proposes a mathematical model to describe the synergetic impact of charge consumption at both electrodes on the capacity retention of full cells. At the same time, “a balanced charge consumption between the cathode and the anode” is proposed as the guidance for the battery design. The manuscript shows that not all the Coulombic efficiency loss will contribute to

permanent capacity loss because the electrolyte decomposition can be compensated through self-discharge. The new concept was mathematically developed and validated with multiple lithium-ion chemistries. This manuscript provides insights into the physical connection of parasitic Coulombic loss at individual electrodes and the remaining capacity in LIB full cells. It is recommended for publishing in Nature Communications, after addressing following concerns:
- In Figure 2, RPT tests before and after OCV aging was conducted to differentiate the capacity retention and the charge retention, both of which were further used to develop the concept of "balanced charge consumption". At the same time in Figure 1c, voltage-capacity slippage was shown for repeated charge/discharge cycling. Is there any implicit, explicit, relationship between the charge consumption during OCV aging and the charge consumption during cycling? Also it is t is recommended to prepare the plot in Fig. 2b with a reference line showing a slope of 1 as mentioned in the text.
- In Scheme 1, LAM and LLI were not fully spelled out anywhere.
- Figure 5d compares the capacity retention of pouch cells using different electrolytes and cycling protocols. It was shown that the cell group incorporating a napping period had a much better capacity retention than the group with the napping period. The performance difference was also reasonably explained with the new concept of balanced charge retention. Will it be universally true that the napping period improve the capacity retention? If not, what will be characteristics of cell chemistries whose capacity retention can be improved with the implementation napping?
-Page 12: If both modes (void filling and continuous) are possibly active in the actual steady leakage current, can fitting with a combination (exp(-at)+t^(-0.5)+i_∞provide a better result? Does this provide any useful change in the calculation of ia and ic?

Reviewer #4

(Remarks to the Author)
I co-reviewed this manuscript with one of the reviewers who provided the listed reports. This is part of the Nature Communications initiative to facilitate training in peer review and to provide appropriate recognition for Early Career Researchers who co-review manuscripts.

Version 1:

Reviewer comments:

Reviewer #1

(Remarks to the Author)
The authors adequately addressed the reviewers' comments and added new data.
I recommend the revised manuscript to be published in nature communications.

Reviewer #2

(Remarks to the Author)
The authors have adressed the open points sufficiently.

Reviewer #3

(Remarks to the Author)
The authors have addressed my comments in the revised manuscript. I recommend its publication at Nat. Comm.

Reviewer #4

(Remarks to the Author)
I co-reviewed this manuscript with one of the reviewers who provided the listed reports. This is part of the Nature Communications initiative to facilitate training in peer review and to provide appropriate recognition for Early Career Researchers who co-review manuscripts.

Open Access This Peer Review File is licensed under a Creative Commons Attribution 4.0 International License, which permits use, sharing, adaptation, distribution and reproduction in any medium or format, as long as you give appropriate credit to the original author(s) and the source, provide a link to the Creative Commons license, and indicate if changes were made.
In cases where reviewers are anonymous, credit should be given to 'Anonymous Referee' and the source.
The images or other third party material in this Peer Review File are included in the article's Creative Commons license, unless indicated otherwise in a credit line to the material. If material is not included in the article's Creative Commons license and your intended use is not permitted by statutory regulation or exceeds the permitted use, you will need to obtain permission directly from the copyright holder.
To view a copy of this license, visit https://creativecommons.org/licenses/by/4.0/

RESPONSES TO REVIEWER COMMENTS

Reviewer #1 (Remarks to the Author):

This paper describes a model that takes into account various reactions in lithium-ion batteries, and predicts the battery life by analyzing the rate of side reactions determined from the self-discharge current during constant potential charging. The reviewer agrees with the authors' claim that the battery life does not correspond to the coulombic efficiency but is related to the current efficiency of the positive and negative electrodes, and convincing evidence is presented in the paper. However, there are many ambiguities regarding the side reactions inside the battery, and more detailed explanations are necessary. Some unclear points are noted, and see my comments below.

The authors appreciate the reviewer's recognition of our findings on the new charge consumption model with prediction ability in this work. The authors are also thankful for many insightful and constructive comments to improve our work. All comments have been carefully addressed as below.

1. Two parameters, ρ and ic/ia, are presented, but no correlation with capacity retention is shown. Is there any relationship between ic/ia in Fig. 5a and capacity retention in c?

Thank you so much for your insightful comment. We have plotted this correlation in Figure R1 as below, showing the strong dependence of ic/ia ratio on capacity retention. It is strongly followed by the Equation 8 in the manuscript and below, where the difference between *ic* (also γ_{Li}^{c}) and *ia* (γ_{Li}^{a}) should be equal or their ratio should be equal to 1 to minimize the capacity loss.

$$Capacity\ loss = \max\left(\gamma_{X^+}^{c}, \gamma_{Y^-}^{a}\right) + |\gamma_{Li}^{c} - \gamma_{Li}^{a}| \quad (8)$$

This also firmly confirms that the balanced *ic* and *ia* plays a significant role in guiding the design of long-life batteries. To address the comment, the Figure R1 was added to the manuscript as Figure 5c. And, a paragraph was also added to the page 16 of the main text as following.

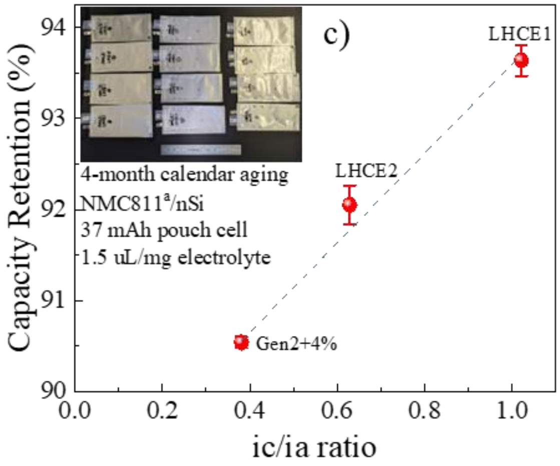

Figure R1. Strong correlation between ic/ia ratio measured via HpLC (from Figure 5a) and capacity retention of large-format pouch cells after 4-month OCV aging.

"Figure 5c exhibits the strong dependence of balanced charge consumptions (i_c/i_a ratio) on the capacity retention of Si-based lithium-ion batteries during calendar aging." on Page 16.

2. The diagram in Fig. 3 seems suspicious and should be drawn more precisely. γe is thought to be an electron passing through an external circuit. If it is an electron in this equation, X -> X+ + e- as shown in the diagram, then γe must be the same as γX. Also, the equation for back insertion should involve the electrons as follows; MO2 + Li+ + e- -> LiMO2, because MO2 + Li+ -> LiMO2 in the diagram does not satisfy the electrical neutrality.

The authors thank the reviewer for insightful comments for potential confusion. To be specific, γ_e^c is a thermodynamic term describing the rate of electron transfer from electrolyte decomposition at the electrode/electrolyte interface, regardless of the external measurements. Given that X^+ is a product of X being oxidized at the cathode/electrolyte interface, not all X^+ will be deposited on the cathode surface. A fraction of the generated X^+ will actually diffuse away from the cathode and into the bulk electrolyte, usually defined as crosstalks, while the rest byproducts of X^+ will be deposited on the cathode surface to involve in the cathode's charge neutrality, denoted as γ_{X+}^c. Therefore, γ_{X+}^c is always a fraction of γ_e^c. To satisfy the charge neutrality, γ_{Li}^c is a charge balancing mechanism, accounting for the portion X^+ diffusing into the bulk electrolyte, following the Equation 5 in the manuscript.

$$\gamma_e^c = \gamma_{X+}^c + \gamma_{Li}^c \tag{5}$$

Worth of emphasizing, γ_{Li}^c could be influenced by cell condition. When cells were placed into a constant-voltage charging mode, the rate of Li back insertion will approach to zero due to no self-discharging in the cathode, and $(\gamma_e^c - \gamma_{X+}^c)$ will be measured as the leakage current during the constant-voltage charging experiments.

Correspondingly, the following revisions have been made to the manuscript.

(1) A sentence as following was added on Page 8 to clarify the physical meaning of γ_e^c.

"Thus, γ_e^a and γ_e^c are thermodynamic terms that describe the rate of electron transfer in electrolyte decompositions at the electrode/electrolyte interfaces, regardless of the external measurements."

(2) A paragraph as following was added on Page 8 to clarify the physical meaning of γ_{X+}^c.

"Meanwhile, electrolyte oxidation or reduction generates positively or negatively charged species and radicals, X^+ or Y^-, respectively. A fraction of the generated X^+ or Y^- diffuse away from the electrode and into the bulk electrolyte, usually defined as crosstalks. The rest byproducts (γ_{X+}^c or γ_{Y-}^a) are deposited on electrode surface for detrimental contributions to permanent capacity loss, like aforementioned detrimental proton-bearing (H^+) species from electrolyte oxidation.[41,50,51] Therefore, the deposited charged species (e.g. γ_{X+}^c) is always a fraction of the charge consumption of electrolyte decomposition (e.g. γ_e^c). The difference between γ_e^c and γ_{X+}^c can describe the contribution of crosstalk, so that the capacity retention of full cells can be roughly calculated using data from half cells"

(3) A paragraph as following was added on Page 9 to clarify the physical meaning of γ_{Li}^c and the external measurements in OCV-RPT tests and leakage current tests.

"Using the cathode boxed in red dotted rectangle as an isolated system, the local charge neutrality must be maintained to accommodate all charge transfer mechanisms. To satisfy the local charge neutrality during OCV aging, γ_{Li}^{c} is a charge balancing mechanism by self-discharging process of the cathode, accounting for the portion X^{+} diffusing into the bulk electrolyte, as the Equation 5.

$$\gamma_e^c = \gamma_{X^+}^c + \gamma_{Li}^c \qquad (5)$$

In OCV-RPT tests, the net of electron transfer across the CEI ($\gamma_e^c - \gamma_{X^+}^c$) equivalently contributes to the charge loss (1-Chr) due to self-discharge process (γ_{Li}^c). Therefore, the byproducts from electrolyte oxidation ($\gamma_{X^+}^c$) partially contributes to the permanent loss of reversible capacity (1-Cr), which is a fraction of the net electron transfer ($\gamma_e^c - \gamma_{X^+}^c$), as shown in Equation 6.

$$\rho_c = \gamma_{X^+}^c \Big/ (\gamma_e^c - \gamma_{X^+}^c) \qquad (6)$$

It is also worth emphasizing that, when cells are at constant-voltage charging mode in HpLC measurements, the rate of Li back insertion (γ_{Li}^c) will approach to zero due to no self-discharging, and the net of electron transfer ($\gamma_e^c - \gamma_{X^+}^c$) will be compensated by the counter Li metal electrode through the external circuit and measured as the leakage current."

3. Please explain in detail the relationship between I(t) in Eq.10-14 and γe. In particular, since γe=0 in the aging state, γX is equal to γLi, because back insertion should be paired with a side reaction to form a local cell. Moreover, the reviewer thinks that the self-discharge current measured in the Li half-cell corresponds to γX at the positive and negative electrodes.

Thank you so much for the comment. Referring to the response to comment #2, γe is a thermodynamic term for the electrons generated from electrolyte decomposition. γ_X is a fraction of positively charged decomposition byproducts to deposit on the electrode surface, while the rest byproducts diffuse away from the electrode into the bulk electrolyte as the crosstalk. The net of charge changes at the electrode (γ_e - γ_X) must be compensated to maintain the local charge neutrality, so the self-discharging during OCV aging (γ_{Li}) or the leakage current ($I(t)$) during constant-voltage holding is the balancing mechanism to satisfy the charge neutrality, as the following relationship:

$$\gamma_e^c - \gamma_{X^+}^c = \gamma_{Li}^c = I(t)$$

About the hypothetical situation when γe=0 in the aging state, there is no electrolyte decomposition for no charge change in the cathode. Thus, the balancing mechanism will not be activated.

4. The authors' claim that the battery life does not correspond to the Coulomb efficiency but is related to the current efficiency of the positive and negative electrodes is also presented in the previous study below, which should be cited.
K. Nakura et al., J. Electrochem. Soc. 158 (2011) A1243–A1250.
J. Xu et al., J. Electrochem. Soc. 162 (2015) A2026–A2035.

R.D. Deshpande et al., J. Electrochem. Soc. 162 (2015) A330–A338.
K. Ariyoshi at al., Electrochemistry 87 (2019) 188–192.

The authors are thankful for the reviewer mentioning some previous studies on finding the disconnection between coulombic efficiency and capacity retention. Those well support our proposed charge consumption mechanisms in this study.

The previous studies (*R.D. Deshpande et al., J. Electrochem. Soc. 162 (2015) A330–A338.; J. Xu et al., J. Electrochem. Soc. 162 (2015) A2026–A2035.*) have been cited on page 2, as below:

"In some exceptional reports, relatively increased oxidative parasitic reactions at the cathode side favored performance improvement in full cells during cycling, e.g. using electrolytes additives, at higher cutoff voltage, or lower anode/cathode capacity ratio.[33-37]"

The studies (*K. Nakura et al., J. Electrochem. Soc. 158 (2011) A1243–A1250.; K. Ariyoshi at al., Electrochemistry 87 (2019) 188–192.*) are mentioned and cited in the manuscript on Page 11.

"Similar findings have been previously reported that improving current efficiency of the cathode and the anode can increase battery capacity retention.[52,53]"

5. In this paper, the authors do not discuss crosstalk reactions, which plays an important role on the side reactions inside the batteries. The reviewer is somewhat skeptical as to why this study was able to predict capacity retention without taking crosstalk responses into account. Please provide some comments on this.

Thank you so much for this insightful comment. Indeed, this is a very important aspect regarding crosstalk. Referring our response to comment #2, the contribution of crosstalk is actually described by the difference between γ_e^c and $\gamma_{X^+}^c$. Therefore, the capacity retention of full cells can be roughly calculated using data from half cells. In this study, we compared the electrochemical performance of the electrodes aged in half cells and the harvested electrodes from full cells during OCV aging into new half cells (Figure S4). Their comparable results indicate that the aging behavior of electrode materials in half cells is well representative to those aged in full cells at a low cutoff voltage of 4.2 V, suggesting minor influence from crosstalk reactions. However, the authors would like to declare that this model captures the majority of charge transfer mechanisms inside a cell, but still missing descriptors accounting for the following contributors. More research is needed to parameterize these contributors to further improve the model's accuracy and adaptability for different conditions.

(1) The fraction of crosstalk species that accept/donate electrons from/to the counter electrode.
(2) The fraction of crosstalk species that deposited on the surface of the counter electrode.
(3) Engineering procedure of anode overhang.
(4) Engineering procedure of N/P ratio.
(5) Engineering control of electrolyte/electrode ratio.
(6) Other aspects not discussed here, such as operation modes in real fields.

Therefore, a paragraph as following was added on Page 6 and Page 8 to clarify the contribution of crosstalk.

"Subsequently, both harvested cathode and anode in half cells were cycled at C/10 for two cycles to show 2.503 mAh and 3.222 mAh discharge capacity, respectively. It is found that the electrochemical

performance of harvested NMC532 and Gr from full cells is almost identical to those 3-month aged in half-cell configurations, 2.503 mAh in NMC532||Li cells and 3.220 mAh in Gr||Li cells (Figures 2c-e, Figure S4)." on Page 6

"Meanwhile, electrolyte oxidation or reduction generates positively or negatively charged species and radicals, X^+ or Y^-, respectively. A fraction of the generated X^+ or Y^- diffuse away from the electrode and into the bulk electrolyte, usually defined as crosstalks. The rest byproducts ($\gamma_{X^+}^{c}$ or $\gamma_{Y^-}^{a}$) are deposited on electrode surface for detrimental contributions to permanent capacity loss, like aforementioned detrimental proton-bearing (H^+) species from electrolyte oxidation.[41,50,51] Therefore, the deposited charged species (e.g. $\gamma_{X^+}^{c}$) is always a fraction of the charge consumption of electrolyte decomposition (e.g. γ_{e}^{c}). The difference between γ_{e}^{c} and $\gamma_{X^+}^{c}$ can describe the contribution of crosstalk, so that the capacity retention of full cells can be roughly calculated using data from half cells." on Page 8

And, a paragraph as following was added on Page 18 to declare the potentially insufficient accuracy of the model.

"Furthermore, this model captures the majority of charge transfer mechanisms inside a cell. There are still many important contributors for battery performance to be included, such as (1) the fraction of crosstalk species that accept/donate electrons from/to the counter electrode and/or deposited on the surface of the counter electrode, (2) the engineering procedures of anode overhang, N/P ratio, and electrolyte/electrode ratio, and (3) other aspects like cell operation modes in real fields. These contributors can be investigated using two recommended dimensionless criteria (i_a/i_c and ρ) in this study to establish their physical descriptors to battery life. It would be vital to establish sufficient dimension and modality of charge consumption mechanism for accurate prediction of various battery chemistries and conditions. "

Reviewer #2 (Remarks to the Author):

The paper under revision "Deciphering Coulombic Loss in Lithium-Ion Batteries and Beyond" provides a study on the physics behind coulombic loss, to ultimately provide a new method to predict lifetime. The topic of lifetime testing and prediction is very relevant as testing takes a considerable amount of time and lifetime is a major contributor to the CO2 footprint as well as the total cost of ownership. In this study, a series of cycle and calendar tests were conducted as a reference for a method that uses voltage hold measurements to calculate characteristics that allow cycle and calendar life to be modeled in 0.5 months for at least 6 months of conventional testing. The results indicate considerable potential in reducing testing time, while still maintaining accurate predictions.
Below are several major and minor points that I think may improve the quality of the paper. Because of these points I would recommend major revisions before publication.

The authors appreciate the positive comments on the fundamental and practical values of physically connected charge consumption model in this study. All comments of both major and minor points have been carefully addressed in the response and revised accordingly in the manuscript. The authors fully agree with the reviewer that this current model roughly captures the major charge consumption mechanism, while bearing the lack of dimensions and accuracy from other factors. Despite this, our work provides very positive evidence that the battery life can be calculated using the accelerated testing data.

Major issues:
1. Implications from the anode overhang

a. When cells are stored at high SOC the geometrical anode overhang is filled which can only partly be extracted again. In lab cells the anode overhang can sometimes have significant contribution to capacity loss and or charge loss

i. Please provide information about the cell design anode and cathode area. Double side coating or only single side coating
ii. Please discuss the impact of anode overhang on the capacity loss and charge loss

Thank you so much for this insightful comment on the influence of anode overhang on capacity loss. The authors agree with the reviewer that anode overhang will certainly play a role in influencing the capacity retention of full cells. Under the scope of this work, the engineering procedure of anode overhang was not taken into account and parameterized in the current model. This important aspect deserves a separate effort to develop a universal descriptor for anode overhang. The following are some facts regarding anode overhang we adopted in this study.

i. CR2032 coin cells for fundamental investigations on coulombic loss and rapid HpLC tests contained 1.4-cm diameter cathode (an area of 1.539 cm^2), 1.5-cm Gr or Si anode (1.767 cm^2). The large-format pouch cells for conceptual demonstration used 3.13 cm x 4.5 cm cathode (14.085 cm^2) and 3.24 cm x 4.6 cm anode (14.904 cm^2). All electrode laminates are single layer coating, and the anode overhang is about 0.5 mm in both coin cells and pouch cells.
ii. The anode overhang very likely brings to some contributions in capacity loss and charge loss. For instance, in Figure 5a, although a linear relationship has been established for the large-range *ic/ia*

charge-balance ratio and the full-cell performance indicator (ρ_f), there is also a small but noticeable deviation among multiple chemistries. This deviation is believed to be the influence of other cell factors like anode overhang, which will require a series of systematic investigations to expand the dimension and modality of charge consumption model. In this work, our work attempts to establish a major connection of charge consumption and battery life in model chemistry and develop the effective criteria using two dimensionless parameters (i_a/i_c and ρ), for the reference of future investigations on different factors.

To boost more attention on the important anode overhang and other factors for the community, this new discussion has been added in the Discussion section on Page 18.

"Furthermore, this model captures the majority of charge transfer mechanisms inside a cell. There are still many important contributors for battery performance to be included, such as (1) the fraction of crosstalk species that accept/denote electrons from/to the counter electrode and/or deposited on the surface of the counter electrode, (2) the engineering procedures of anode overhang, N/P ratio, and electrolyte/electrode ratio, and (3) other aspects like cell operation modes in real fields. These contributors can be investigated using two recommended dimensionless criteria (i_a/i_c and ρ) in this study to establish their physical descriptors to battery life. It would be vital to establish sufficient dimension and modality of charge consumption mechanism for accurate prediction of various battery chemistries and conditions."

In addition, more information was added in the Methods section on Page 20 to clarify the cell geometry used for this study, as following:

"CR2032 coin cells for fundamental investigations on coulombic loss and rapid HpLC tests contained 1.4-cm diameter cathode (an area of 1.539 cm^2), 1.5-cm diameter anode (1.767 cm^2), or 1.6-cm diameter and 100-µm thick Li metal foil. The 37-mAh large-format pouch cells for conceptual demonstration used 3.13 cm x 4.5 cm cathode (14.085 cm^2) and 3.24 cm x 4.6 cm anode (14.904 cm^2). All electrode laminates are single layer coating, and the anode overhang is about 0.5 mm in both coin cells and pouch cells."

2. Capacity retention and charge retention are very low for the investigated systems

The authors appreciate the major concern on the performance of our model system. After a careful review of existing literature, it is noted that some literature-reported or commercial NMC/Gr cells have employed electrolyte engineering with novel solvents or additives, electrode modification, and undisclosed strategies for commercial confidentiality to show the improved electrochemical performance. These chemistry deviations cause difficult reproducibility and validation of NMC/Gr performance by the academic community to approve/disapprove this new concept. To facilitate the delivery of the new concept, we purposely selected a simple model system without further modification in electrolyte or electrode. It is the BASELINE to be easily repeated in the experiment by peers. For example, we have confirmed that our cycling results are comparable to the reported cycling performance of NMC532/Gr cells using the same BASELINE electrolyte without any additive, while some electrolyte additives (FEC or VC) in the

BASELINE electrolyte exhibited excellent performance improvements. Thus, it is not a surprise that the performance data shown in this work is slightly below the topnotch industrial cells, which contains proprietary technologies. Therefore, the authors believe that the reported cell chemistry is representative of state-of-the-art lithium-ion chemistry and is proper for use as a model system.

We have addressed the major concerns as following:

a. i.e. figure 2 b) a capacity retention of < 90% after 3 months for the full cell at 4.2 V is found please compare this to reported data from commercial cells and comment on how the obtained performance of the investigated cells might influence the outcome with regard to state-of-the-art cells.

About the comparison of state-of-the-art calendar aging results with our results (NMC/Gr cells lose about 12% capacity in OCV aging at 4.3V for 3 months at 30 oC)

- Three different commercial cells using NCA, NMC, and LFP cathode and Gr anode lose about 10% when aging the cells at 4.2V for 10 months at 40 oC. (*J. Electrochem. Soc., 163(9): A1872-A1880 (2016)*). Although their results show better performance, no disclosed electrolytes was provided for those commercial cells from Panasonic, Sanyo, and A123 cell manufacturers. Also, their half-cell validation used 2%VC additive in the electrolyte. It is well known VC has excellent chemical stability to significantly improve cell performance.
- Some comparable results for calendar aging have been reported. NMC/Gr cells lose 18% capacity in OCV aging at 4.18V for 200 days at 40 oC. The electrolyte is constituted of 1 M LiPF6 salt in a non-disclosure organic solvent without any additive. (*Journal of Power Sources 239 (2013) 572-583*). NMC/Gr cells using the electrolyte of 1.2M LiPF6 in EC/EMC (3/7 by weight) with 10% FEC additive exhibited about 77% capacity retention after 2160-hr or 3-month calendar aging at 4.1V at 30 oC (*Journal of The Electrochemical Society, 2022 169 050531*)
- The state-of-the-art NMC/Li cell results are comparable or not better to what we reported in this study (2% loss at 4.3V for 3 months at 30oC). For instance, Dufek et.al. reported that NMC pouch cells using lithium metal anode showed 3.66% loss for a calendar life of 5 months at 4.4V at 25 oC (*Energy Storage Materials, 65: 103147, 2024*). In addition, Zhang et. al. reported that NMC/Li cells using a locally high concentration electrolyte lose about 22% reversible capacity in 200 days when aged at 4.4V and 30 oC. (*Energy Environ. Sci., 2023, 16, 1548*).
- In addition, the data reported in this work have a higher capacity fade rate is simply because we were measuring the capacity fade rate at 100% SOC (4.3V) while "better data" on state-of-the-art chemistries were generally collected at a low SOC. An extremely long calendar life can be easily and simply achieved by aging the cells at low SOC (<4.0V). It has been a common wisdom of this community that extended calendar life at low SOC is ascribed to the slower parasitic reactions at low SOC. (*Journal of Energy Storage 26 (2019) 100900; J. Phys. Chem. C 2017, 121, 21865−21876*).

b. i.e. figure 1 b) same here for the capacity retention after cycling 1000 cycles led to 73% SOH

About the comparison of state-of-the-art cycling results with our results (NMC532/Gr cells show 73% capacity retention after 1000 cycles)

- 70.5-79% capacity retention was reported for NMC532/Gr full cells at 4.2V after 1000 cycles, using the baseline electrolyte of LiPF6 and EC/EMC(3:7) without any additive. These reported results are in good agreement with our results in this study. (*Journal of Power Sources 342 (2017) 846-852;*

Journal of Power Sources 515 (2021) 230429; Journal of Physics and Chemistry of Solids 200 (2025) 112614) These literature reports have been cited in the manuscript on Page 3:
"Agreeing well with the open literature using the baseline chemistry (also summarized in Table S1),[42-44] this model chemistry had excellent electrochemical stability, maintaining about 73% of its initial reversible capacity after 1000 charging/discharging cycles (Figure 1b)."

- In comparison, electrolyte additives (FEC or VC) in the baseline electrolyte or the commercial proprietary electrolyte can significantly improve the capacity retention to 86-88% at 4.2V after 1000 cycles. (*Journal of The Electrochemical Society, 2022 169 010501; Journal of The Electrochemical Society, 165 (11) A2732-A2740 (2018); Journal of The Electrochemical Society, 164 (6) A1037-A1049 (2017); Journal of Power Sources 498 (2021) 229884*)

c. figure 1 b) please show the results from multiple cells. we found strong deviations (100%) for this in our data from LiFun Cells

Figure R2 shows the repeatable long-term performance of three NMC532/Gr full cells using the baseline electrolyte without additive, and the error bars are the standard deviations of measured capacity and CE. This Figure R2 has also been updated for Figure 1b on Page 5 in the manuscript.

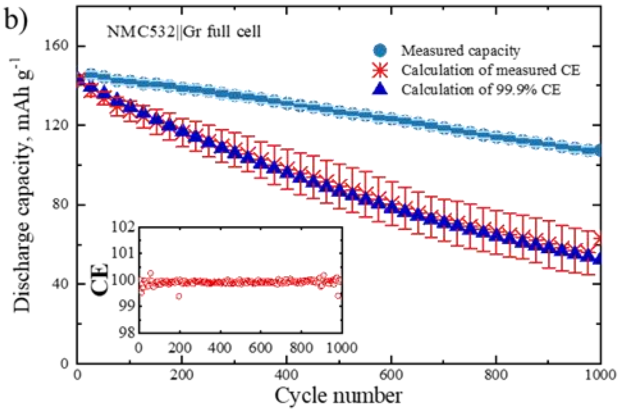

Figure R2. Three repeating NMC532||Gr cells in the voltage range of 2.5-4.2 V at a constant current of C/2 (~ 1.5 mA), showing the heavy overestimation of irreversible charge inventory loss using measured and reported CE values.

The LiFun cells and other reported literature of NMC532/Gr chemistry have been summarized in Table R1 to compare with our results. It is noted that our results are comparable with the reported cycling performance of NMC532/Gr chemistry using the baseline chemistry without any modification. Particularly, our results showed the capacity fading rate of 0.027 %/cycle, within the range of 0.02-0.119 %/cycle in the reported literature. In comparison, the modification using electrolyte additive and/or surface coating on cathode can significantly improve the cycling performance. With these improvements, the capacity fading rate can dramatically reduce to 0.005-0.01 %/cycle, much lower than the baseline chemistry with significant separation. Therefore, the strong deviation is ascribed to the different modification chemistries for NMC532/Gr cells. It is also our intention to perform investigations on the baseline chemistry without modification for ease of reproducibility to approve/disapprove the new charge consumption concept. Table R1 was also added in the Supporting Information for Table S1.

Table R1. Summary of cycling performance of NMC532/Gr cells in various chemistries.

Manufactor	Electrolyte	Modification	Voltage V	Temperature °C	Cycling Performance	Capacity Fading %/cycle	Ref
CAMP	1.2M LiPF6 in EC/EMC (3:7)	none	4.2	R.T.	73% - 1000 cycles	0.027	this study
lab	1.2 M LiPF6 in EC:DEC (3:7)	none	4.2	30	80% - 1000 cycles	0.020	1
lab	1.2M LiPF6 in EC/EMC (3:7)	none	4.2	-	70.5% - 500 cycles	0.119	2
lab	1.2M LiPF6 in EC/EMC (3:7)	none	4.2	30	74% - 1000 cycles	0.026	3
LiFun	1M LiPF6 in EC/EMC (3:7)	none	4.4	40	90% - 300 cycles	0.033	4
LiFun	1.5M of LiPF6 in EC/EMC/DMC (25:5:70)	none	4.3	40	80% - 500 cycles	0.040	5
LiFun	1.5M of LiPF6 in EC/EMC/DMC (25:5:70)	none	4.3	40	80% - 300 cycles	0.067	6
LiFun	1.2M LiPF6 in EC/EMC (3:7)	2%FEC additive	4.2	40	96% - 400 cycles	0.010	7
LiFun	1.5M LiPF6 in EC/DMC (3:7)	2% VC additive	3.8	40	95% - 1000 cycles	0.005	8
LiFun	1.1M LiPF6 in EC/EMC (3:7)	2%VC + 1%DTD additives	4.3	40	95% - 900 cycles	0.006	9
LiFun	1.5M LiPF6 in EC/EMC (3:7)	2%VC + 1%DTD additives	4.2	40	95% - 1000 cycles	0.005	10
LiFun	1.2M LiPF6 in EC/EMC (3:7)	Al2O3 coating, VC211 additives	4.2	20	90% - 1000 cycles	0.010	11

References:

1. Journal of Power Sources 342 (2017) 846-852
2. Journal of Physics and Chemistry of Solids 200 (2025) 112614
3. Journal of Power Sources 515 (2021) 230429
4. Journal of The Electrochemical Society, 164 (7) A1534-A1544 (2017)
5. Journal of The Electrochemical Society, 2024 171 110510
6. Journal of The Electrochemical Society, 2022 169 110513
7. Journal of The Electrochemical Society, 165 (5) A1027-A1037 (2018)
8. Journal of The Electrochemical Society, 2022 169 050512
9. Journal of The Electrochemical Society, 2021 168 020532
10. Journal of The Electrochemical Society, 165 (3) A626-A635 (2018)
11. Journal of The Electrochemical Society, 165 (13) A3195-A3204 (2018)

d. how would you explain such deviations?

It is noted that such deviations are strongly correlated to the different modification chemistries for NMC532/Gr cells. According to the model in this study, the $Capacity\ loss = \max\left(\gamma_{X^+}^{c}, \gamma_{Y^-}^{a}\right) + |\gamma_{Li}^{c} - \gamma_{Li}^{a}|$. A stable chemistry can improve the performance by following two terms in this relationship. For instance, a surface coating of Al2O3 or the preferential decomposition of the FEC additive can stabilize the cathode surface to avoid the damage from the acidic proton-bearing species. This will bring down the first term for irreversible active material loss. In addition, as newly proposed in this study, the stable chemistry should be able to balance out the charge consumption between the cathode and the anode for minimal inventory loss. It is known that VC or FEC additive can generate a stable SEI at the surface of anode material, which is critical for bringing down the charge consumption from parasitic reactions at the anode.

e. please compare your found self-discharge of around 20% (figure 2 c ~0.5 mAh/2.5 mAh) to other publications on calendar aging

NMC/Gr cells using the electrolyte of 1.2M $LiPF_6$ in EC/EMC (3/7 by weight) with 3% FEC additive exhibited 15% charge consumption after first 4-week calendar aging at 4.1V at 30 oC (e.g. 0.375 mAh loss in 2.5 mAh) and then 10% charge consumption after second 4-week (about 0.2 mAh loss). It should be also noted that FEC additive typically stabilizes chemical stability for improved performance. In addition, NMC/Si cells using the same electrolyte had 20% coulombic loss after first 4 week (e.g. 0.5 mAh in 2.5 mAh). Please refer to Figure 4 in *Frontiers in Batteries and Electrochemistry 2 (2023): 1308127.*

A relatively stable LFP/Gr cell chemistry using the electrolyte of 1.2M $LiPF_6$ in EC/EMC (3/7 by weight) with 10% FEC additive exhibited over 20% charge consumption (i.e. Qrev in Equation 5 and Figure 8) after over 2160-hour or 3-month calendar aging at 3.335V at 30 oC. Please refer to *Journal of The Electrochemical Society, 2022 169 050531.*

Herein, through these comparisons, our results of 20% coulombic loss during longer duration of 3 months and higher voltage at 4.3V (figure 2c) is comparable to the reported literature.

i. please provide OCV vs time data for the OCV tests.

Figure R3 presents the time evolutions of open-circuit voltage in multiple cell chemistries, which has been added in Supporting Information in Figure S6.

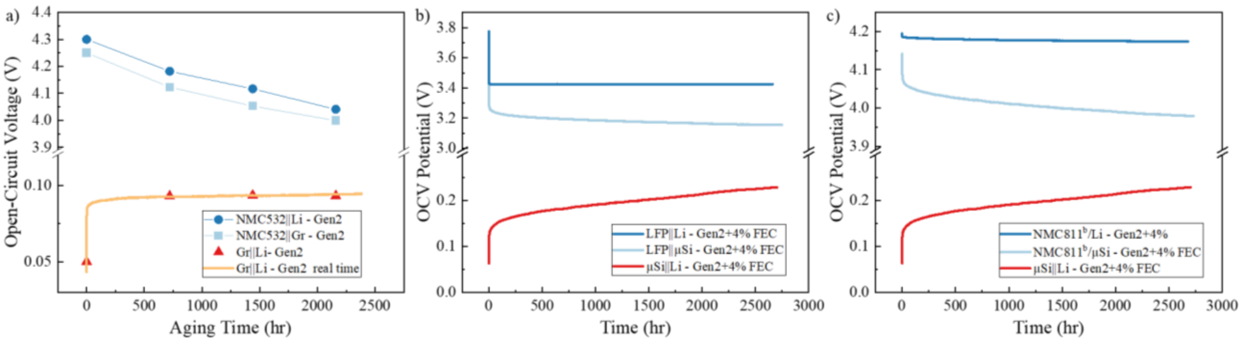

Figure R3. The time evolution of open-circuit voltage in NMC532||Gr, LFP||µSi, and NMC811||µSi full cells and their corresponding half cells during calendar aging over 3 months.

f. please highlight the temperature under which each experiment was conducted

The coin cells were stored in 30 oC temperature chamber for calendar aging experiments and leakage current measurements. The cycling tests were conducted at room temperature.

Several paragraphs were added in the Methods sections on Page 21-22 to clarify the testing conditions.

"All reported OCV-aging data in this study were collected from three repeating coin cells or two repeating large-format pouch cells at 30 °C." in ***Open-circuit-voltage (OCV) aging.***

"All reported HpLC data in this study were collected from three repeating coin cells at 30 °C." in ***High-precision leakage current measurement.***

"All reported cycling data in this study were collected from three repeating coin cells or two repeating large-format pouch cells at room temperature." in ***Routine electrochemical characterization.***

3. Can the authors please discuss on how the li metal will influence the half cell results, I was thinking on crosstalk especially for the impact of different electrolytes.

Thank you for this insightful comment on the influence of crosstalk reactions. As discussed in the Response to the Reviewer 1's comment #5, the contribution of crosstalk was implicitly embedded into the term ($\gamma_e^c - \gamma_{X^+}^c$). It is noted that electrolyte decomposition generates electrons (e^- or γ_e^c) and charged species (X^+). A fraction of X^+ species diffuses away from the electrode into the bulk electrolyte as the crosstalk, while the rest species is deposited on electrode with charge ($\gamma_{X^+}^c$). The net charge transfer to electrode ($\gamma_e^c - \gamma_{X^+}^c$) and the balancing charge by Li back insertion (γ_{Li}^c) or the leakage current (*I(t)*) are relevant to the crosstalk species. In addition, as shown in Figure S4, the electrodes aged in half cells exhibited comparable electrochemical behavior with the harvested electrodes from full cells after 3-month OCV aging in new half cells. This indicates minor influence from crosstalk reactions for this stable cell chemistry at a low cutoff voltage of 4.2 V. The authors fully agree that the crosstalk fraction of accepting/donating electrons or depositing at the counter electrode certainly deserves a new descriptor to parameterize these contributions to improve the dimension and accuracy of the charge consumption model. Despite this, this model demonstrates that the majority of charge transfer mechanisms can be calculated to predict battery life, using the accelerated testing data.

In this work, lithium counter is preferred for measuring leakage current in half cells for several reasons.

(1) Leakage current was measured at semi-steady state when the current value is generally smaller than C/1000 (<1 μA) for equilibrium Li^+ concentration gradient at the extended time. Therefore, the interface impedance due to aggravated parasitic reactions and crosstalk influence at lithium surface has insignificant impact on leakage current.
(2) The electrochemical potential of lithium redox reactions occurs at 0 V for both lithium stripping/plating. This ensures accurate control on the potential of the working electrode to measure the leakage current at the extended time.
(3) Also, Li metal counter anode in half cells provides necessary charge inventory. When gaining/donating electrons (*I(t)*) from/to the external circuit, Li counter anode correspondingly plates/strips Li^+, which can satisfy all net charge consumption at the working electrode ($\gamma_e^c - \gamma_{X^+}^c$ or γ_{Li}^c) without changing the potentials of Li counter anode and working electrode. This is a self-reaction of stripping/plating Li for external charge consumption, which is additional to the reductive parasitic reactions at the Li metal surface.
   To be clearer, the reductive parasitic reactions at Li metal anode (γ_e^{Li}) are exclusively dependent on thermodynamic reactivity of Li metal and electrolyte, independent of external electrons (*I(t)*). As long as Li inventory is excessive, Li counter anode in half cells can self-compensate all intrinsic charge consumption for reductive parasitic reactions, and will not affect external electron transfer (*I(t)*). The intrinsic charge transfer mechanisms at Li counter anode are the electron release (γ_e^{Li}) to reduced electrolyte species, the deposited charged species ($\gamma_{Y^-}^{Li}$) on Li metal, and the diffusing/crosstalk charged species (γ_e^{Li} - $\gamma_{Y^-}^{Li}$) into the bulk electrolyte. The net intrinsic charge consumption at Li metal (γ_e^{Li} - $\gamma_{Y^-}^{Li}$) is all satisfied by self-disharging/stripping Li (γ_{Li}^{Li}) at the constant redox potential. It is also noted that the total charge inventory change at Li counter anode in half cells should be sum of intrinsic and external charge compensation ($\gamma_{Li}^{Li} + \gamma_{Li}^c$ or γ_{Li}^{Li} + *I(t)*).

4. Statistics and error discussion

a. Please provide standard deviation and sample size for each reported data set.

b. Please provide voltage and current accuracy of the Keithley 2401 source meter

Thank you so much for this constructive comment. All reported data in this study were collected from three repeating coin cells for OCV-aging tests, cycling tests, and HpLC measurements, and two repeating pouch cells for conceptual demonstration. The sample sizes for routine cycling tests, OCV-aging tests, and HpLC measurements have been mentioned in the Method section on Page 21-22. Their standard deviation has been provided to the reported data in Figure 1-5 in the manuscript.

Keithley 2401 source meter has a voltage accuracy of 100 μV for the range of 20 V and, for current measurements, has 10 nA accuracy for 1 mA range and 100 pA accuracy for 10 μA range. (https://www.testequipmenthq.com/datasheets/KEITHLEY-2401-Datasheet.pdf). This specification has been added in the method section on Page 22.

5. Please provide information on the napping procedure in the method section:

a. for how long was the rest phase

b. at which SOC

c. for how often?

The detailed "napping" cycling protocol has been added in the method section on Page 21, including the OCV resting duration, the starting SOC before "napping", the every-cycle frequency, as below. It should be noted that the "napping" procedure is particularly developed for approving the significance of global charge compensation mechanism. It should also cautiously implement this "napping" procedure in the practical applications, regarding to balancing ic/ia characteristics and electrolyte mass consumption in targeting battery chemistry.

"The cycling protocol with the "napping" procedure (denoted CC-CV-OCV test) in Figure 5d-e first followed 10 formation cycles at C/10 in 2.5-4.2 V and then followed a sequence of constant-current (CC) charging to 4.2V at C/10, constant-voltage (CV) holding at 4.2 V for 1 hour, and the open-circuit-voltage (OCV) "napping" or resting from 4.2 V for 1 hour, and then constant-current discharge to 2.5 V at C/10 in every cycle till completing 100 cycles. The "control" cycling protocol followed the above procedure, except no "napping" OCV step before discharging."

6. The paper suggests a correlation of the charge balance parameter (ic/ia) and the cell performance indicator (ρf), please provide for all tested cell chemistries in Figure 5 a) the OCV-RPT data as well as cycling data. Give a quantitative analysis on which ic/ia range is beneficial and in which manner this is needed for further validation of the proposed method.

Thank you so much for these constructive comments. We have provided the voltage profiles and statistical OCV-RPT and HpLC data for Figure 5a) of multiple Si-based lithium-ion chemistries in Figure R4-R7. Only selected chemistries (Gen2+4%FEC, LHCE 1, and LHCE 2) were tested for cycling performance in two repeating large-format pouch cells, as shown in Figure 5d-e in the manuscript. Those Figure R4-R7 were also added to Figure S9-12 in the Supporting Information and referenced in the manuscript accordingly.

Ideally, the ic/ia descriptor of balancing charge consumption should be equal to 1 to get all charge inventory compensated for good performance. Considering the nature of the electrochemical reactions as Tafel equation, the rate of electrolyte decomposition and charge consumption is strongly dependent on the working/holding potential. Regarding some practical situations, 3C electronic products and electrical vehicles might be partially charged below the target 100% SOC to begin working or resting. Thus, the ic/ia should be also evaluated for entire working potential range to seek for the minimal difference between ic and ia. In addition, it should be noted in this study that $capacity\ loss = \max(\gamma_{X^+}^{c}, \gamma_{Y^-}^{a}) + |\gamma_{Li}^{c} - \gamma_{Li}^{a}|$. A balanced ic/ia only minimizes the second term. The cell performance is still governed by the first

thermodynamics term $\max(\gamma_{X^+}^{c}, \gamma_{Y^-}^{a})$, which can be minimized by optimizing the individual electrode/electrolyte interface.

The balancing charge mechanism can be also tested from the charge loss (I*t) during OCV aging at the same duration, to further validate of the proposed ic/ia method.

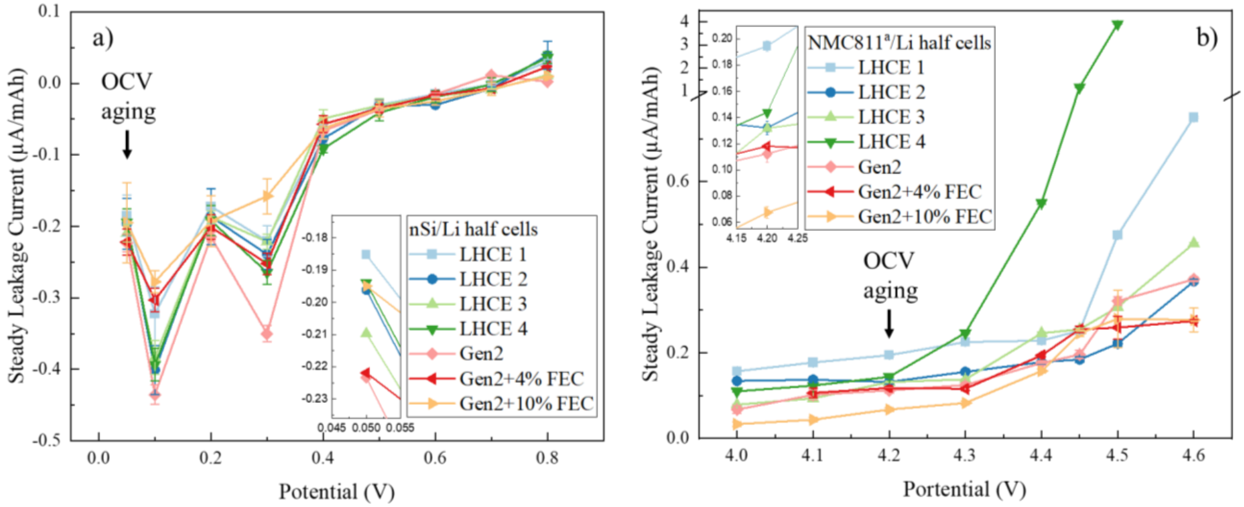

Figure R4. The rate of a) reductive parasitic reactions (i_a) at Si anode and b) oxidative parasitic reactions (i_c) at NMC cathode as a function of their corresponding voltage range via HpLC measurments. The ic/ia ratio of each chemistry in the Figure 5a was calculated for their approximate voltage (i.e. 0.05V at Si anode and 4.2V at NMC cathode) as OCV aging begins. The error bar is the standard deviation of three repeating cells. Added to Figure S9 in the Supporting Information.

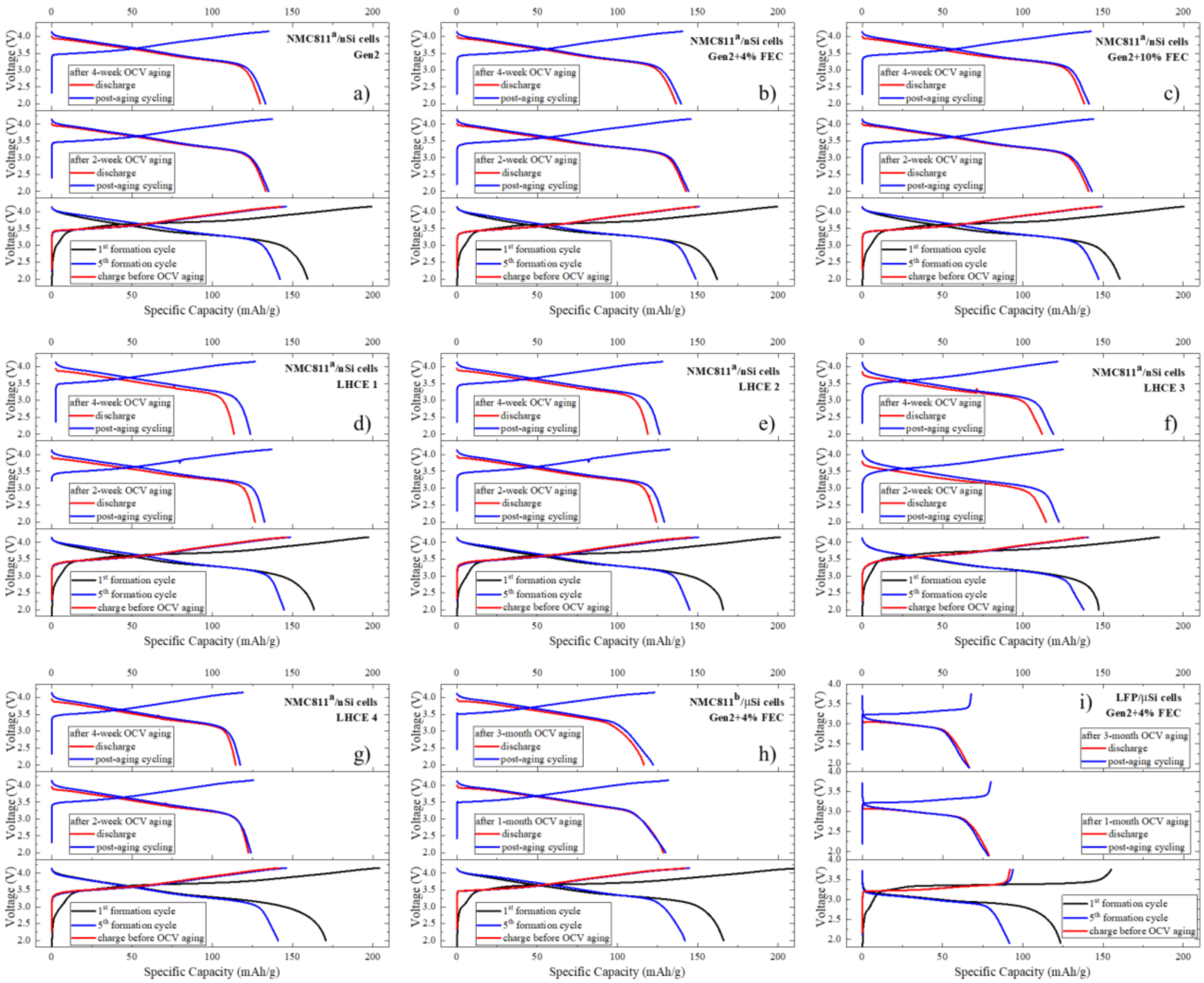

Figure R5. Voltage profiles of multiple Si-based lithium-ion battery chemistries during calendar aging tests, each from one of three repeating coin cells. Added to Figure S10 in the Supporting Information.

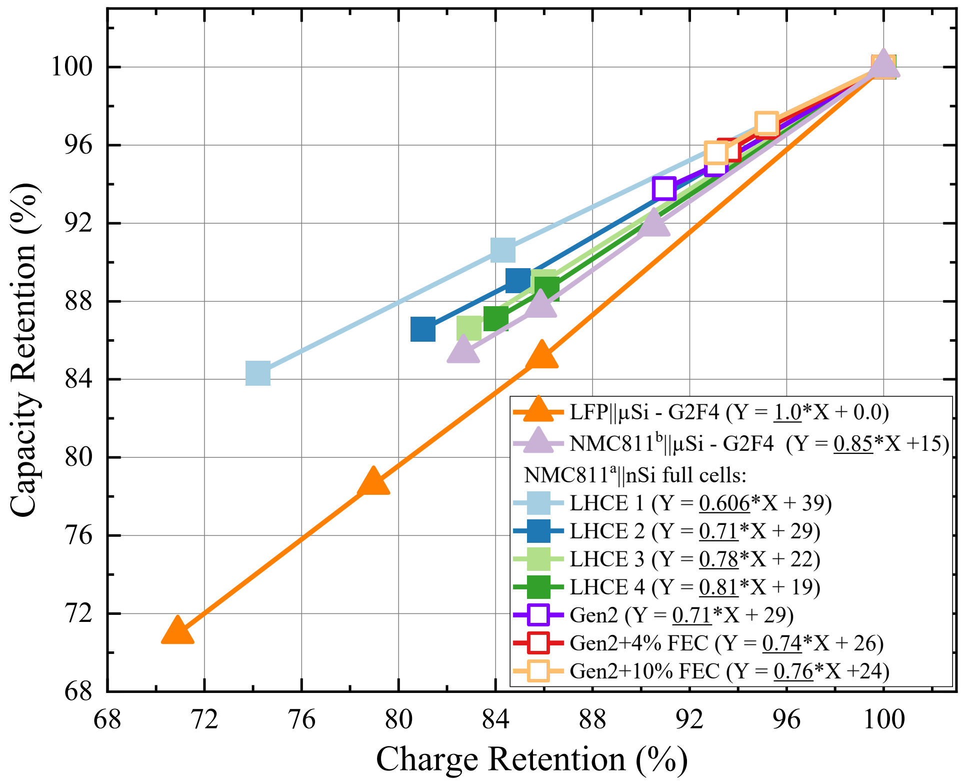

Figure R6. Linear relationship between the measured capacity retention and the charge retention for multiple Si-based lithium-ion battery chemistries during calendar aging. Each data point represents the statistical data of three repeating coin cells. Added to Figure S11 in the Supporting Information.

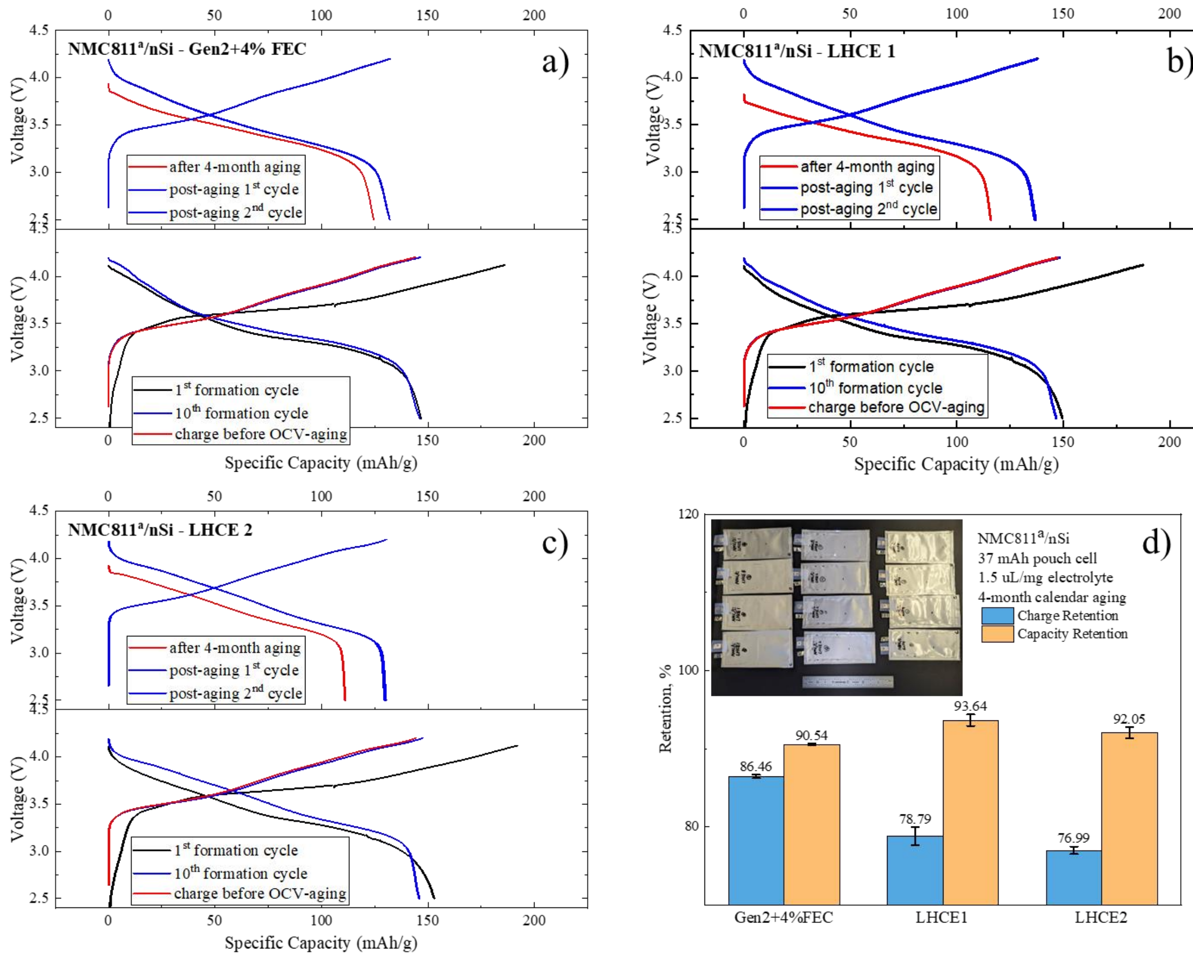

Figure R7. (a-c) Voltage profiles of 37-mAh large-format NMC811[a]/nSi pouch cells using a) Gen2+4% FEC, b) LHCE 1, and c) LHCE 2 electrolytes, before and after 4-month calendar aging. (d) The comparison of charge retention and capacity retention during calendar aging. The error bar is the standard deviation of two repeating pouch cells. Added to Figure S12 in the Supporting Information.

7. In my opinion, the standard aging test is more likely to be done by evaluating the discharge capacity/energy over the cycle as shown in Figure 5 d). Please discuss the advantages of your methods including the accuracy when considering measurement errors and compare it to the direct measurement as performed in Figure 5 d) when the same period of testing time is used in both methods. To my understanding, in the standard cycling test I will get direct quantitative results, while the new method is intriguing, it only results in parameters that correlate qualitatively with performance.

Thank you so much for this comment to improve our work. The “napping” procedure involving OCV resting step in Figure 5d was implemented intentionally to approve the concept of global charge compensation mechanism in the single chemistry (Gen2+4%FEC), in order to discard other possible influences using the ic/ia-optimized electrolyte chemistries (LHCE 1). Although the “napping” OCV step at the end of charging can aggravate electrolyte decomposition and chemical degradation in electrode material, the performance can still be improved by significantly compensating the loss of lithium inventory. Both “napping” procedure in single chemistry and the ic/ia-optimized chemistry elaborate the importance of self-regulating charge-

compensation in suppressing the loss of lithium inventory for improved performance, even without the use of economically expensive pre-lithiation process. Nevertheless, it should be very cautious to implement the "napping" procedure in practical applications, due to aggravated battery degradation during the extended time at high voltage. As mentioned in the manuscript, the wise choice is suggested to use the ic/ia-optimized chemistry (e.g. LHCE1 electrolyte) for naturally balanced charge consumptions to improve performance. In addition, the "napping" procedure is also far from practical testing and diagnosis as a new protocol in battery management system (BMS) that requires restricted investigations and quantitative evaluations.

To resolve the possible misunderstanding and misuse of "napping" procedure, the explicit description and some extended discussion have been added in the manuscript, on Page 17.

"In order to examine the global charge compensation and discard other possible influences using different electrolytes, a "napping" procedure of OCV step was intentionally incorporated at the end of charging process after 10 formation cycles to permit certain self-discharging or re-lithiation at the cathode. Compared with regular charging/discharging procedure, the same cell chemistry (NMC811[a]/nSi with Gen2+4% FEC) takes proper "napping" of self-discharging in the cathode to recover Li inventory and exhibits significant improvement in full cell performance (Figure 5d,e). This reveals that global charge inventory compensation is favorable for sustaining stable performance from the continuous loss of Li inventory. It should be cautiously noted that, nevertheless, the "napping" procedure intentionally implemented for conceptual proving on global charge compensation mechanisms might not be suitable for practical applications, due to aggravated electrolyte depletion and electrode degradation for an extended time at high voltage. Certainly, the wisest choice is balancing the charge consumptions between the cathode and the anode to minimize the loss of Li inventory and improve cycling performance, as shown in the optimal performance using the balanced i_c/i_a LHCE1 electrolyte (Figure 5d). Both the i_c/i_a-optimized chemistry and "napping" procedure have experimentally validated that the global charge inventory compensation underlies the self-regulation mechanism to suppress the loss of lithium inventory and improve battery life."

Minor issues:

8. Writing style. The clarity of the document could be improved:

The authors are grateful for many suggestions to improve writing and understanding in the manuscript.

a. Limiting sentences to maximum of 3 lines (i.e. page 3 "This investigation uncovered two dimensionless and measurable coulombic descriptors to quantitatively connect with battery capacity in physically meaningful manners: one being ρ, which is defined as the "detrimental" ratio that partial by products of parasitic reactions are responsible for irreversible capacity fading of electrode materials, and the other, ic/ia, defined as the "balanced" ratio that coulombic consumptions between cathode and anode should be balanced to globally compensate the usable inventory in full cells."

The revision of shorter sentences has been made in the Introduction section on Page 3.

"This investigation uncovered two dimensionless and measurable coulombic descriptors to quantitatively connect with battery capacity in physically meaningful manners, i.e. the "detrimental" ratio as ρ and the "balanced" ratio as i_c/i_a."

c. Avoid judgmental wording like "Large discrepancy" – "heavy overestimation"

The judgemental wording has been revised in the manuscript, as below:

"The apparent inconsistency underlies the overestimation of irreversible charge inventory loss towards the capacity fading in Equation 1, rather than the random errors from inaccurate measurements and non-optimized chemistries." on Page 4

"**Figure 1. Notable inconsistency between the measured coulombic loss and capacity fading.**" in Figure 1 caption on Page 5

"(b) Cycling discharge capacity of NMC532||Gr cell in the voltage range of 2.5-4.2 V at a constant current of C/2 (~ 1.5 mA), showing the overestimation of irreversible charge inventory loss using measured and reported CE values." in Figure 1 (b) caption on Page 5

"(c) The voltage-capacity slippage of NMC532||Gr cell during cycling, presenting non-equivalence of reversible capacity and the accumulated coulombic consumption by parasitic reactions." in Figure 1 (c) caption on Page 5

d. Remove filling phrases

We have removed the unnecessary filling phrases and also rephrased the sentences throughout the manuscript, including "like", "well", "very", and "highly", as highlighted in change tracking.

d. When results are presented it is helpful to give the found results in text and elaborate how the conclusion was drawn. (i.e. "The NMC532||Gr full cells after OCV-RPT tests for 3 months were dissembled to harvest NMC532 and Gr electrodes and prepare them in half cells using fresh Li metal anode and electrolyte. It is found that the electrochemical performance of harvested NMC532||Li and Gr||Li cells is identical to those aged in half-cell configuration (Figures 2c-e, Figure S4)." Here only a conclusion is given and the reader has to look at the data, assess by him/herself how that conclusion was drawn.

We have revised the commented text in the manuscript on Page 6 and also made other revisions for better understanding as the highlight in the change tracking.

"The NMC532||Gr full cells after OCV-RPT tests for 3 months were discharged to 2.5 V and then dissembled to harvest NMC532 and Gr electrodes and prepare them in half cells using fresh Li metal anode and electrolyte. The harvested NMC532 in half cells were first discharged to cathode's 0% SOC at 2.5V vs Li/Li^+ at C/10 current rate, showing a 0.278 mAh discharging capacity (about 11% of initial capacity) due to lithium vacancy in the cathode (Figure 2d). In comparison, the harvested Gr in half cells had no discharging/delithiation capacity to anode's 0% SOC at 1.5 V vs Li/Li^+ (Figure 2e). This 11% capacity loss could represent the loss of lithium inventory during their full-cell operation. Subsequently, both harvested cathode and anode in half cells were cycled at C/10 for two cycles to show 2.503 mAh and 3.222 mAh discharge capacity, respectively. It is found that the electrochemical performance of harvested NMC532 and Gr from full cells is almost identical to those 3-month aged in half-cell configurations, 2.503 mAh in NMC532||Li cells and 3.220 mAh in Gr||Li cells (Figures 2c-e, Figure S4). This confirms that the aging behavior of electrode materials in half cells is representative to those aged in full cells."

9. The authors state several times that the community would rely on coulomb efficiency when developing new chemistries. I do not agree here. The main testing carried out is cycle life testing focusing on discharge capacities. This is not to state that the found quantification and modeling of the charge loss is less relevant, however, I find the motivation a little exaggerated.

Thank you for this comment. The authors agree that the current evaluation for new chemistries greatly relies on the discharge capacity, which is the most direct description to battery life. However, the charging/discharging tests require very lengthy evaluation duration to gain enough data and modality for accurately screening multi-chemistries and predicting battery life. In this context, the development of some coulomb descriptors, including CE, is highly demanded by the community for rapid evaluation, as some recent perspectives on CE for accurate performance evaluation (Nature energy 5.8 (2020): 561-568.; Nature Energy 6.10 (2021): 951-960.). They have also openly discussed the long-standing challenges of implementing CE's quantitative analysis for accurate battery performance evaluation, suggesting CE as a semi-quantitative performance indicator. Our findings in this work can provide the key insight on the ignored physical connection between CE and battery capacity, to enable the accurate and quantitative performance evaluation using CE.

Reviewer #3 (Remarks to the Author):

Quantitative relationship between the charge consumption at the electrode/electrolyte interface and the battery performance is crucial for understanding the performance degradation mechanism and providing guidance for rational design of high performance batteries. The authors identified a method to compare how OCV aging affects capacity loss and charge loss differently with high-precision static leakage current measurements of the anode and cathode (ic/ia) and used it to predict the capacity loss very accurately. Focusing on different charge consumption rate at the cathode and the anode, this manuscript proposes a mathematical model to describe the synergetic impact of charge consumption at both electrodes on the capacity retention of full cells. At the same time, "a balanced charge consumption between the cathode and the anode" is proposed as the guidance for the battery design. The manuscript shows that not all the Coulombic efficiency loss will contribute to permanent capacity loss because the electrolyte decomposition can be compensated through self-discharge. The new concept was mathematically developed and validated with multiple lithium-ion chemistries. This manuscript provides insights into the physical connection of parasitic Coulombic loss at individual electrodes and the remaining capacity in LIB full cells. It is recommended for publishing in Nature Communications, after addressing following concerns:

The authors are thankful for our fundamental contributions on understanding coulombic loss and the developed prediction model being well recognized and positively commented. We also thank for many insightful comments to improve our work, and have addressed them as below.

1. In Figure 2, RPT tests before and after OCV aging was conducted to differentiate the capacity retention and the charge retention, both of which were further used to develop the concept of "balanced charge consumption". At the same time in Figure 1c, voltage-capacity slippage was shown for repeated charge/discharge cycling. Is there any implicit, explicit, relationship between the charge consumption during OCV aging and the charge consumption during cycling? Also it is t is recommended to prepare the plot in Fig. 2b with a reference line showing a slope of 1 as mentioned in the text.

It is first noted the oxidative parasitic reactions at the cathode tend to generate electrons, no matter charging or discharging in cathode. The measured coulombs during charging are the sum of the cathode capacity that also provides electrons (C) and the coulombic consumption by oxidative parasitic reactions (Q), i.e. C + Q. Differently, the measured coulombs during discharging are the difference of cathode capacity and the parasitic coulombic consumption, i.e. C - Q. The voltage-capacity slippage in Figure 1c accumulates the difference between charging and discharging coulombic measurements over 1000 repeating cycles, (C+Q) – (C-Q) = 2Q. In comparison, charge consumption during OCV aging directly measures the self-discharge coulombic reduction by oxidative parasitic reactions during extended OCV time in one single cycle. If extended OCV time (1 month = 720 hr) is much longer than the charging time (10 hr), the charge consumption during OCV aging can be roughly regarded as Q. Herein, the charge consumption during OCV aging is approximately half of charge consumption during cycling, as shown for the cycling capacity slippage (Figure 1c) much larger than OCV charge loss (Figure 2b).

In addition, Figure R8 plots the reference line for a slope of 1 to show the apparent deviation between measured capacity retention and charge retention on Page 7, which has been updated in Figure 2b.

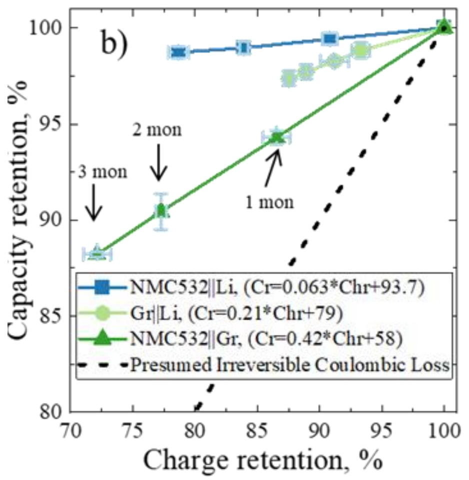

Figure R8. ….. b) Linear relationship between the capacity retention and the charge retention for NMC532||Li, Gr||Li and NMC532||Gr cells. A reference for the slope of 1 based on the prevailingly believed irreversible coulombic loss is provided in the black dash line, showing apparent deviation from the realistic situations. The error bar of each data point is the standard deviation of measured capacity retention or charge retention in three repeating cells.

The text for Figure 2b has also been updated accordingly on Page 6 for the comparison of measured slopes and presumed slope.

"However, their linear slopes of less than 1 show apparent deviation from the presumed irreversible coulombic loss in Equation 1, suggesting that only parts of parasitic coulombic consumption are responsible for capacity fading.

2. In Scheme 1, LAM and LLI were not fully spelled out anywhere.

We have made the revision in the Introduction section on Page 2.

"The measured coulombic loss in LIBs during operation is prevailingly believed as the capacity fading mainly due to parasitic reactions at the electrode/electrolyte interfaces (EEIs), including the loss of active materials (LAM) and the loss of lithium inventory (LLI) or charge inventory.[11,12]"

3. Figure 5d compares the capacity retention of pouch cells using different electrolytes and cycling protocols. It was shown that the cell group incorporating a napping period had a much better capacity retention than the group with the napping period. The performance difference was also reasonably explained with the new concept of balanced charge retention. Will it be universally true that the napping period improve the capacity retention? If not, what will be characteristics of cell chemistries whose capacity retention can be improved with the implementation napping?

Thank you for this comment. The "napping" procedure in Figure 5d was deliberately implemented to validate the concept of a global charge compensation mechanism for the unbalanced ic/ia chemistry (e.g. Gen2+4%FEC). Due to voltage manipulation in cycling tests, the electrons generated in sluggish oxidative

parasitic reactions at the cathode are completely consumed by the faster reductive parasitic reactions at the anode, leading to no charge inventory compensation from the cathode. In this context, a “napping” procedure incorprates an OCV resting step at the end of charge to permit self-discharging in the cathode to gain Li inventory from the electrolyte, thus compensating certain Li loss at the anode and sustaining globably usable Li inventory during discharging in full cells. Theoretically, this “napping” procedure become less favorable for the ic/ia-balanced chemistry, because Li inventory can be intrinsically zeroed from the equal generation at the cathode and consumption at the anode. In addition, this napping procedure for approving fundamental concept should be very cautious to implement in practical applications of ic/ia-unbalanced chemistries, due to aggravated battery degradation during the extended time at high voltage. For sake of battery design, it is recommended to use ic/ia-optimized chemistry (such as the LHCE1 electrolyte) for naturally balanced charge consumption to enhance performance.

Clear description and for “napping” procedure has been added in the manuscript, please refer to Page 17.

4. Page 12: If both modes (void filling and continuous) are possibly active in the actual steady leakage current, can fitting with a combination (exp(-at)+t^(-0.5)+i_∞provide a better result? Does this provide any useful change in the calculation of ia and ic?

Thank you for this insightful comment. We have implemented the hybrid model fitting of combining void-filling and continuous growth models with the weighting factors of A1 and A2, respectively, as shown in Figure R9 below. Apparently, the continuous-growth part in the hybrid model occupies a dominant role in fitting leakage current profiles during the static voltage-holding process. In other words, incorporating void filling model will generate a larger deviation to overestimate the rate of parasitic reactions.

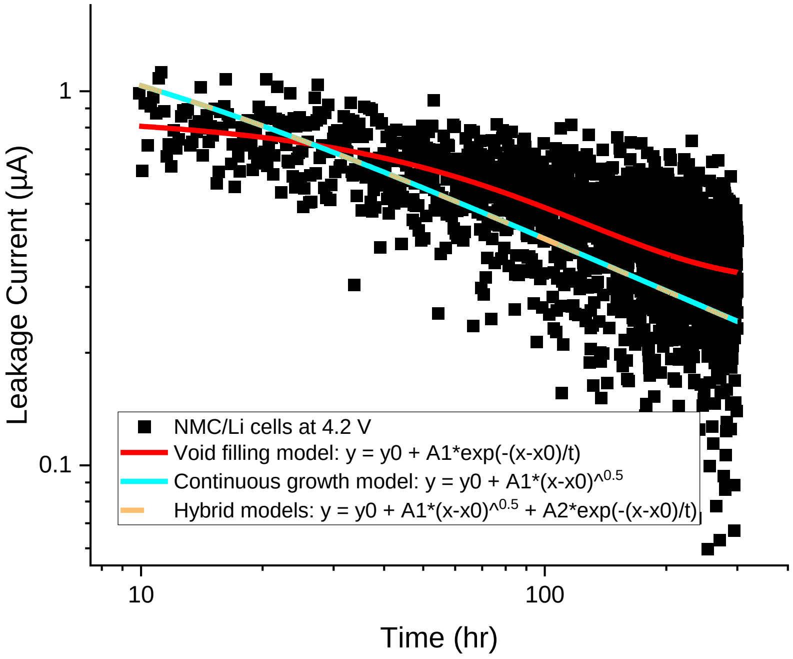

Figure R9. Comparison of fitting HpLC data of NMC||Li half cells at 4.2V using void-filling model, continuous-growth model, and their hybrid model.

Reviewer #4 (Remarks to the Author):

I co-reviewed this manuscript with one of the reviewers who provided the listed reports. This is part of the Nature Communications initiative to facilitate training in peer review and to provide appropriate recognition for Early Career Researchers who co-review manuscripts.

The authors are also thankful for your review and comments together with another reviewer.

RESPONSES TO REVIEWER COMMENTS

Reviewer #1 (Remarks to the Author):

The authors adequately addressed the reviewers' comments and added new data.

I recommend the revised manuscript to be published in nature communications.

The authors are thankful Reviewer #1 for reviewing our manuscript and accepting all revisions with the recommendation to publish.

Reviewer #2 (Remarks to the Author):

The authors have adressed the open points sufficiently.

The authors are grateful Reviewer#2 for suggesting many open points for our improvements and providing a positive comment to support our revised manuscript for publication.

Reviewer #3 (Remarks to the Author):

The authors have addressed my comments in the revised manuscript. I recommend its publication at Nat. Comm.

The authors deeply appreciate Reviewer#3 for reviewing our revisions and giving the recommendation to publish.

Reviewer #4 (Remarks to the Author):

I co-reviewed this manuscript with one of the reviewers who provided the listed reports. This is part of the Nature Communications initiative to facilitate training in peer review and to provide appropriate recognition for Early Career Researchers who co-review manuscripts.

The authors appreciate Reviewer#4 for co-reviewing our manuscript and providing comments to improve our work for a better publication.
